# Physical, Mental, and Social Characteristics Associated With Happiness in Individuals With Schizophrenia in Japan: A Cross‐Sectional Study

**DOI:** 10.1002/npr2.70045

**Published:** 2025-09-04

**Authors:** Junna Hattori, Masaaki Matsunaga, Yupeng He, Kenji Sakuma, Taro Kishi, Shinichi Tanihara, Nakao Iwata, Atsuhiko Ota

**Affiliations:** ^1^ Department of Public Health School of Medicine, Fujita Health University Toyoake Japan; ^2^ Department of Psychiatry School of Medicine, Fujita Health University Toyoake Japan; ^3^ Department of Public Health School of Medicine, Kurume University Kurume Japan

**Keywords:** cross‐sectional studies, epidemiology, happiness, schizophrenia, sex differences

## Abstract

**Objective:**

To examine the characteristics associated with happiness in Japanese individuals with schizophrenia.

**Methods:**

A self‐reported online survey was conducted in 2022 among individuals aged 20–75 years, including 223 and 1776 individuals with and without schizophrenia, respectively. We used a modified Poisson regression to assess the factors associated with happiness by calculating the age‐ and sex‐adjusted prevalence ratios (PRs). We examined within‐schizophrenia group differences by age and sex strata, and compared these stratified PRs between groups with and without schizophrenia.

**Results:**

Among participants with schizophrenia, happiness was significantly associated with self‐rated health status (PR = 1.75), *Ikigai* (PR = 5.02), depressive symptoms (PR = 0.43), perceived stress (PR = 0.52), cognitive social capital (PR = 2.07), structural social capital (PR = 1.70), social support (PR = 2.40), close friends (PR = 1.88), close relatives (PR = 2.34), and a cohabiting partner (PR = 1.57). Within the schizophrenia group, sex differences were significant for cognitive social capital (men: PR = 3.45; women: PR = 1.43) and cohabiting partners (men: PR = 2.26; women: PR = 1.25), whereas no significant age differences were found. Factors demonstrating a stronger association in participants with schizophrenia than in those without schizophrenia included: *Ikigai* (with, PR = 5.02; without, PR = 2.91), cognitive social capital (with, PR = 2.07; without, PR = 1.49), and structural social capital (with, PR = 1.70; without, PR = 1.24).

**Conclusion:**

Happiness in individuals with schizophrenia is associated with physical, mental, and social factors, with social factors exhibiting sex‐related differences.

## Abstract 2

This study identified key characteristics of happiness in Japanese individuals with schizophrenia by comparing them to individuals without schizophrenia and analyzing internal subgroup differences. The findings are twofold: first, factors like a sense of purpose (Ikigai) and social capital were more strongly associated with happiness in the schizophrenia group than in those without schizophrenia. Second, within the schizophrenia group, these social factors showed significant variation, with associations being stronger for men than for women.

## Graphical abstract

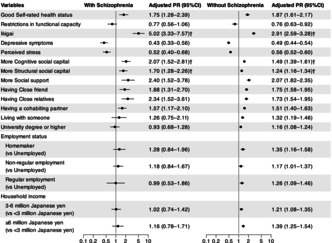

## 1 Introduction

Happiness, encompassing affective well‐being (feelings of joy and pleasure), eudaimonic well‐being (sense of meaning and purpose in life), and evaluative well‐being (life satisfaction), has emerged as a major focus in health research [1]. While extensive research has examined happiness in the general population [1, 2], the understanding of happiness among individuals with schizophrenia remains limited across different cultural contexts, including in Japan. This is an important area of investigation, as evidence suggests that schizophrenia is associated with alterations in emotional experiences [3] and a sense of purpose [4]; however, interestingly, many individuals with schizophrenia report satisfaction with their lives despite associated symptoms and functional impairments [5].

A growing body of research has identified various factors associated with happiness in individuals with schizophrenia. Clinical factors, such as low stress levels [6] and high functional capacity [7] have been shown to be positively associated with happiness. Mental and psychological factors, including health‐related quality of life and positive psychological traits (such as resilience, optimism, and personal mastery), have also demonstrated positive associations with happiness [6]. Social factors, such as loneliness, have been associated with lower levels of happiness [8]. In contrast, factors such as illness duration, positive symptoms, cognitive function, educational background, and physical health have demonstrated no statistically significant association with happiness [6]. Negative symptoms demonstrated mixed associations across different measures of happiness [6, 9]. Although studies in the general population have demonstrated associations between happiness and various social factors, such as social capital [10, 11], income [12, 13], and interpersonal relationships [14], these relationships remain largely unexplored in individuals with schizophrenia.

Previous studies have considered individuals with schizophrenia as a homogeneous group when examining happiness. However, the social circumstances (e.g., employment and marriage rates) [15, 16] of individuals with schizophrenia vary considerably by sex, suggesting that factors associated with happiness may also differ when considering sex differences. Furthermore, age may affect the perceptions of and factors related to happiness among individuals with schizophrenia as the symptoms reported vary between younger and older patients [17, 18]. Specifically, younger patients often exhibit more prominent positive symptoms, whereas older patients tend to show more negative symptoms and cognitive decline. Moreover, whether these factors are differently related to happiness in individuals with and without schizophrenia remains unclear. Examining these demographic variations could help identify the unique characteristics of happiness in schizophrenia.

Given this diversity in demographic backgrounds, we hypothesized two key relationships based on previous findings. First, considering sex differences in social circumstances [15, 16], we hypothesized that associations between social factors and happiness would vary by sex. Second, given age‐related clinical variations [17, 18], we hypothesized that associations between mental factors and happiness would differ across age groups.

To examine these hypotheses, this study aimed to examine the characteristics of happiness among individuals with schizophrenia through two complementary approaches to identify the potential effect of heterogeneity of demographic factors on happiness among individuals with schizophrenia. First, we explored whether the factors associated with happiness varied according to demographic characteristics within the schizophrenia group by analyzing the differences between the sex and age groups. Second, we investigated whether the patterns of happiness‐associated factors varied between individuals with and without schizophrenia across age and sex.

## 2 Methods

### 2.1 Design and Participants

This study was conducted as a secondary analysis of our previous study that estimated the prevalence of schizophrenia in Japan [19, 20, 21, 22]. Data collection was performed in February 2022 through Rakuten Insight (https://insight.rakuten.co.jp/), an Internet survey company with approximately 2.2 million registered participants at that time. Informed consent was obtained from all the study participants via the Internet.

The study population comprised two groups. We initially identified 5584 potential participants who self‐reported schizophrenia from the Rakuten Insight Disease Panel [23], which is a subset of the Rakuten Insight Panel.

The key eligibility criteria for individuals with schizophrenia were as follows: Participants were required to have a current diagnosis of schizophrenia and a documented history of auditory hallucinations. Those with a history of stimulant use, alcohol dependence, or symptom onset at age ≥ 60 years were excluded.

The preliminary survey response rate was 58.3% (3256/5584). Of the 3256 respondents, 2740 were excluded for currently having psychiatric conditions other than schizophrenia. Among the remaining 516 participants, 276 were excluded for not reporting auditory hallucinations. From the remaining 240 participants, 17 were further excluded for stimulant use, alcohol dependence, or auditory hallucination onset at age ≥ 60 years. Finally, 223 participants who met all eligibility criteria were included in the analysis as participants with schizophrenia (overall inclusion rate: 4.0% of the initial pool).

For individuals without schizophrenia, we recruited 28 000 participants from two established population surveys: the Japan Society and New Tobacco Internet Survey and the Japan COVID‐19 and Society Internet Survey [24, 25, 26]. Of the 6656 respondents, 1776 participants meeting the eligibility criteria were included in the analysis as participants without schizophrenia.

The key eligibility criteria for individuals without schizophrenia were as follows. Participants were required to have no history of mental illness or auditory hallucinations. Those with a history of stimulant use, alcohol dependence, or psychiatric treatment were excluded. Detailed screening procedures and questions addressed to individuals with and without schizophrenia have been described in previous studies [19, 20].

### 2.2 Variables

The outcome measure of this study was self‐reported happiness. Participants were asked to assess their level of happiness by responding to the question, “How happy do you feel?” with one of the following four options: (1) “very happy,” (2) “happy,” (3) “neither,” or (4) “not happy.” For analytical purposes, responses were dichotomized: participants who selected “very happy” or “happy” were categorized as happy, while those who chose “neither” or “not happy” were categorized as not happy.

The explanatory variables were selected based on factors previously associated with happiness in the general population. These variables were categorized into three domains: physical, mental/psychological, and social factors.

Physical factors included the body mass index (BMI), functional capacity restriction, and self‐rated health status (SRHS). BMI was classified into three categories: (1) < 18.5 kg/m^2^, (2) 18.5 to 24.9 kg/m^2^, and (3) ≥ 25.0 kg/m^2^. Functional capacity restrictions were evaluated using the scale of independence in daily living for older individuals with disabilities, established by the Ministry of Health, Labour, and Welfare [27]. Participants who reported no physical disabilities were coded as having “no restrictions,” while those reporting any form of disability were coded as having “restrictions.” The SRHS, which reflects not only an individual's physical health and functional status but also their well‐being [28], was dichotomized as good or poor.

Mental and psychological factors included Ikigai, depressive symptoms, and perceived stress. Ikigai, a Japanese term signifying a positive reason for living or the basis of one's life [29], was coded as “present” or “absent.” Depressive symptoms were assessed using the 11‐item Center for Epidemiological Studies Depression (CES‐D) Scale [30, 31], with scores ≥ 8 indicating the presence of depressive symptoms. Perceived stress was measured using the 4‐item Perceived Stress Scale (PSS‐4) [32]. As there is no universally established clinical cutoff for the PSS‐4, we defined “high stress” as a score greater than 7, which was the median score observed in participants without schizophrenia in this study.

Social factors included social capital, social support, interpersonal relationships, marital status, living status, employment status, household income, and educational background. Social capital was assessed using cognitive (trust in others) and structural (community participation) social capital. Cognitive social capital was measured using the Social Capital Integrated Questionnaire (SC‐IQ), with scores ≥ 2 indicating high cognitive social capital [33]. Structural social capital was determined by participation in community organizations, self‐help groups, charities, volunteer groups, or religious groups. Participants who reported attending these meetings were classified as having high structural social capital. Social support was evaluated using the ENRICHD Social Support Instrument (ESSI), with scores ≥ 17 indicating high social support [34, 35]. Interpersonal relationships were assessed by the presence of close friends or relatives. Those who reported having at least one close friend or relative were coded as “having close friends/relatives.” Marital status was categorized as “having a cohabiting partner” or “not having a cohabiting partner.” Living status was classified as “living with someone” or “living alone.” Employment status was categorized into four groups: (1) unemployed, (2) homemaker, (3) non‐regular employment, and (4) regular employment. Household income was grouped into three categories: (1) < 3 million yen, (2) 3–6 million yen, and (3) > 6 million yen. Educational background was classified into two categories: university degree or higher, and less than a university degree. Detailed descriptions of all the assessment measures have been provided in our previous study [19].

### 2.3 Analysis

Comparisons between participants with and without schizophrenia were performed for each variable. *T*‐tests were used for continuous variables, and Fisher's exact tests were used for categorical variables.

Associations between happiness and various factors across domains were examined using a modified Poisson regression analysis. For all the factors, prevalence ratios (PRs) of happiness and 95% confidence intervals (CIs) adjusted for sex and age were calculated. As a sensitivity analysis, we performed ordinal logistic regression using the original four‐category happiness scale to assess the robustness of our findings obtained from the dichotomized outcome.

First, we examined whether factors associated with happiness varied according to the demographic characteristics, specifically among participants with schizophrenia. Stratified analyses were conducted within the schizophrenia group based on age and sex. Based on prior studies on happiness in young individuals with schizophrenia and Japanese legal age classifications [5, 36], age was dichotomized into two groups: 20 to 34 years and ≥ 35 years. We performed analyses for each demographic subgroup of participants with schizophrenia (men vs. women, age < 35 vs. age ≥ 35) separately and then formally tested the differences in associations between these subgroups. Differences in the associations between the age groups, and males and females, within the schizophrenia group were evaluated using *p*‐values of interaction terms (*P*_int_) between age or sex and each variable in the modified Poisson regression models. These interaction terms were calculated by including a product term between the subgroup indicator (sex or age) and the variable of interest. A *p*‐value (*P*_int_) of less than 0.05 was considered indicative of a statistically significant difference in the PRs between the demographic subgroups among participants with schizophrenia.

Second, we compared the strength of the associations between the participants with and without schizophrenia to identify whether the factors associated with happiness varied according to the schizophrenia status. These between‐group comparisons were performed across the entire study population and separately within each sex category (comparing men with schizophrenia to men without schizophrenia, and women with schizophrenia to women without schizophrenia) and within each age group (comparing younger individuals with schizophrenia to younger individuals without schizophrenia, and older individuals with schizophrenia to older individuals without schizophrenia). The difference in the PRs between participants with and without schizophrenia was evaluated using the *p*‐value (*P*_diff_) derived from the Wald chi‐squared test statistic, which directly quantifies the magnitude of the difference between the two PRs. The Wald statistic was calculated as follows: [b_1_—b_2_]^2^/{[SE(b_1_)]^2^ + [SE(b2)]^2^}, where b_1_ and b_2_ are the coefficients for each variable for individuals with and without schizophrenia, respectively, and SE(b_1_) and SE(b_2_) are the corresponding standard errors in the age‐ and sex‐adjusted models, respectively. This statistic follows a 1‐degree of freedom Wald chi‐squared distribution. A *p*‐value (*P*_diff_) less than 0.05 was considered indicative of a statistically significant difference in the PRs between participants with and without schizophrenia.

The subgroup analyses were considered exploratory in nature. Therefore, no adjustments for multiple comparisons were made for these analyses, and their results should be interpreted as hypothesis‐generating. The web‐based survey employed in this study featured forced‐choice responses, where participants were required to answer each question to proceed. Consequently, there were no missing data for the variables included in the present analysis, and imputation techniques were not required.

The significance level was set at *p* < 0.05 (two‐tailed). All the analyses were performed using the R version 4.4.1 software.

## 3 Results

Table 1 presents the characteristics of the participants with and without schizophrenia categorized by sex. Participants with schizophrenia demonstrated significant differences in multiple domains compared to those without schizophrenia. Physically, participants with schizophrenia had higher BMI values, a higher prevalence of being overweight, a greater proportion of restrictions in functional capacity, and a higher rate of poor SRHS. Psychologically, participants with schizophrenia are less likely to report *Ikigai* (a sense of purpose in life) and tend to experience more depressive symptoms and stress. Socially, the schizophrenia group demonstrated lower cognitive social capital and was less likely to have close friends. Additionally, women with schizophrenia reported receiving less social support and having fewer close relatives than women without schizophrenia. Furthermore, participants with schizophrenia were less likely to have a cohabiting partner. Women with schizophrenia, however, were less likely to live alone, while no such difference was observed among men. Socioeconomically, participants with schizophrenia had a higher unemployment prevalence, lower prevalence of regular employment, lower household income, and lower educational attainment than participants without schizophrenia.

**TABLE 1:** Characteristics of subjects, stratified by schizophrenia and sex.

Variables	Men			Women		
	Participants with schizophrenia (*n* = 115)	Participants without schizophrenia (*n* = 801)	*p* ^a^	Participants with schizophrenia (*n* = 108)	Participants without schizophrenia (*n* = 975)	*p* ^a^
Happiness			< 0.001			< 0.001
Happy	42 (37%)	494 (62%)		56 (52%)	697 (71%)	
Not happy	73 (63%)	307 (38%)		52 (48%)	278 (29%)	
Age	47.6 (8.6)^b^	48.0 (13.5)^b^	0.651	44.1 (9.8)^b^	41.5 (12.8)^b^	0.014
Body mass index (kg/m^2^)	26.4 (5.1)	23.4 (3.5)	< 0.001	24.2 (5.3)	20.9 (3.2)	< 0.001
Body mass index category			< 0.001			< 0.001
≥ 25	61 (53%)	222 (28%)		42 (39%)	90 (9.2%)	
18.5–24.9	52 (45%)	539 (67%)		56 (52%)	692 (71%)	
< 18.5	2 (1.7%)	40 (5.0%)		10 (9.3%)	193 (20%)	
Self‐rated health status			< 0.001			< 0.001
Good	64 (56%)	665 (83%)		51 (47%)	833 (85%)	
Poor	51 (44%)	136 (17%)		57 (53%)	142 (15%)	
Restrictions in functional capacity			< 0.001			< 0.001
Present	45 (39%)	59 (7.4%)		44 (41%)	45 (4.6%)	
Absent	70 (61%)	742 (93%)		64 (59%)	930 (95%)	
*Ikigai*			< 0.001			< 0.001
Present	49 (43%)	500 (62%)		48 (44%)	628 (64%)	
Absent	66 (57%)	301 (38%)		60 (56%)	347 (36%)	
Depressive symptoms			< 0.001			< 0.001
Present	80 (70%)	251 (31%)		86 (80%)	351 (36%)	
Absent	35 (30%)	550 (69%)		22 (20%)	624 (64%)	
Perceived stress			< 0.001			< 0.001
High	88 (77%)	397 (50%)		83 (77%)	490 (50%)	
Low	27 (23%)	404 (50%)		25 (23%)	485 (50%)	
Cognitive social capital			0.046			0.014
More	45 (39%)	397 (50%)		50 (46%)	575 (59%)	
Less	70 (61%)	404 (50%)		58 (54%)	400 (41%)	
Structural social capital			0.782			0.168
More	16 (14%)	123 (15%)		18 (17%)	117 (12%)	
Less	99 (86%)	678 (85%)		90 (83%)	858 (88%)	
Social support			0.328			0.012
More	76 (66%)	566 (71%)		75 (69%)	784 (80%)	
Less	39 (34%)	235 (29%)		33 (31%)	191 (20%)	
Close friend			0.007			0.024
Present	59 (51%)	519 (65%)		72 (67%)	750 (77%)	
Absent	56 (49%)	282 (35%)		36 (33%)	225 (23%)	
Close relatives			0.437			0.001
Present	79 (69%)	580 (72%)		71 (66%)	780 (80%)	
Absent	36 (31%)	221 (28%)		37 (34%)	195 (20%)	
Marital status			< 0.001			0.003
Having a cohabiting partner	21 (18%)	463 (58%)		41 (38%)	519 (53%)	
Not having a cohabiting partner	94 (82%)	338 (42%)		67 (62%)	456 (47%)	
Living status			0.546			0.018
Living alone	22 (19%)	177 (22%)		11 (10%)	191 (20%)	
Living with someone	93 (81%)	624 (78%)		97 (90%)	784 (80%)	
Educational background			< 0.001			0.003
University degree or higher	47 (41%)	479 (60%)		31 (29%)	427 (44%)	
Less than a university degree	68 (59%)	322 (40%)		77 (71%)	548 (56%)	
Employment status			< 0.001			< 0.001
Unemployed	58 (50%)	119 (15%)		36 (33%)	66 (6.8%)	
Homemaker	3 (2.6%)	5 (0.6%)		29 (27%)	257 (26%)	
Non‐regular employment	40 (35%)	188 (23%)		38 (35%)	309 (32%)	
Regular employment	14 (12%)	489 (61%)		5 (4.6%)	343 (35%)	
Household income			< 0.001			< 0.001
< 3 million Japanese yen	61 (53%)	148 (18%)		47 (44%)	211 (22%)	
3–6 million Japanese yen	40 (35%)	280 (35%)		40 (37%)	375 (38%)	
≥ 6 million Japanese yen	14 (12%)	373 (47%)		21 (19%)	389 (40%)	

^a^ *p*‐values were calculated by two‐sample *t*‐test for continuous variables and Fisher's exact test for categorical variables.

^b^ Continuous variables are presented as mean (SD).

**FIGURE 1:**
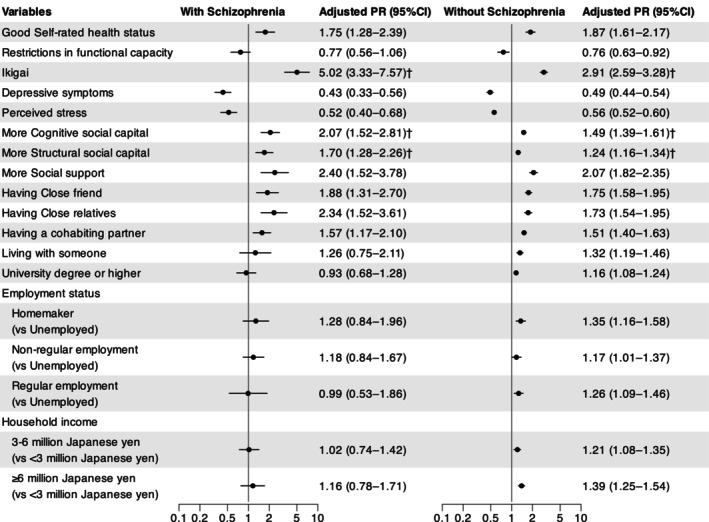
Sex‐ and age‐adjusted prevalence ratios and 95% confidence intervals for happiness in participants with and without Schizophrenia. Adjusted PR, Sex‐ and Age‐adjusted prevalence ratio. ^†^ *p* < 0.05 for the difference in PRs (*P*_diff_) between participants with and without schizophrenia.

Figure 1 shows the age‐ and sex‐adjusted PRs and 95% CIs for participants with and without schizophrenia. Significant associations with happiness were observed in both groups for SRHS, Ikigai, depressive symptoms, perceived stress, cognitive social capital, structural social capital, social support, close friends, close relatives, and marital status. In contrast, the associations were not significant for functional capacity restrictions, living status, educational background, employment status, and household income in participants with schizophrenia. The magnitude of associations varied significantly between individuals with and without schizophrenia for having Ikigai (with schizophrenia: PR = 5.02, 95% CI = 3.33–7.57 vs. without schizophrenia: PR = 2.91, 95% CI = 2.59–3.28, *P*_diff_ = 0.012), more cognitive social capital (with schizophrenia: PR = 2.07, 95% CI = 1.52–2.81 vs. without schizophrenia: PR = 1.49, 95% CI = 1.39–1.61, *P*_diff_ = 0.042), and more structural social capital (with schizophrenia: PR = 1.70, 95% CI = 1.28–2.26 vs. without schizophrenia: PR = 1.24, 95% CI = 1.16–1.34, *P*_diff_ = 0.035).

**FIGURE 2:**
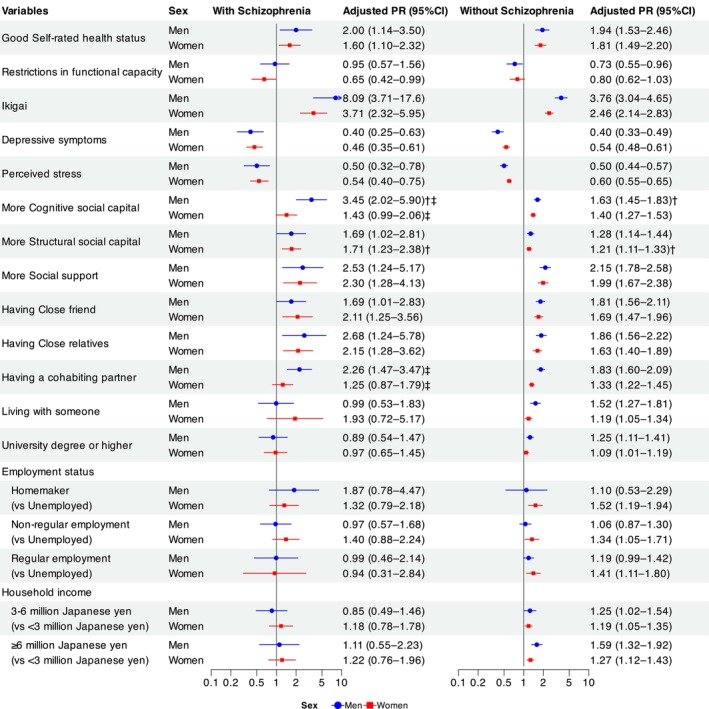
Age‐adjusted prevalence ratios and 95% confidence intervals for happiness in participants with and without Schizophrenia, Stratified by Sex. Adjusted PR, Age‐adjusted prevalence ratio. ^†^ *p* < 0.05 for the difference in PRs (*P*_diff_) between participants with and without schizophrenia. ^‡^ *p* < 0.05 for the interaction (*P*_int_) between sex and each variable among participants with schizophrenia.

Figure 2 shows the PRs and 95% CIs for participants with and without schizophrenia, stratified by sex. Cognitive social capital in men was more strongly associated with happiness in participants with schizophrenia than in those without schizophrenia (with schizophrenia: PR = 3.45, 95% CI = 2.02–5.90 vs. without schizophrenia: PR = 1.63, 95% CI = 1.45–1.83, *P*_diff_ = 0.007). When comparing men and women within the schizophrenia group, cognitive social capital (men: PR = 3.45, 95% CI = 2.02–5.90 vs. women: PR = 1.43, 95% CI = 0.99–2.06, *P*_int_ = 0.008), having a cohabiting partner (men: PR = 2.26, 95% CI = 1.47–3.47 vs. women: PR = 1.25, 95% CI = 0.87–1.79, *P*_int_ = 0.039) was more strongly associated with happiness in men than in women.

**FIGURE 3:**
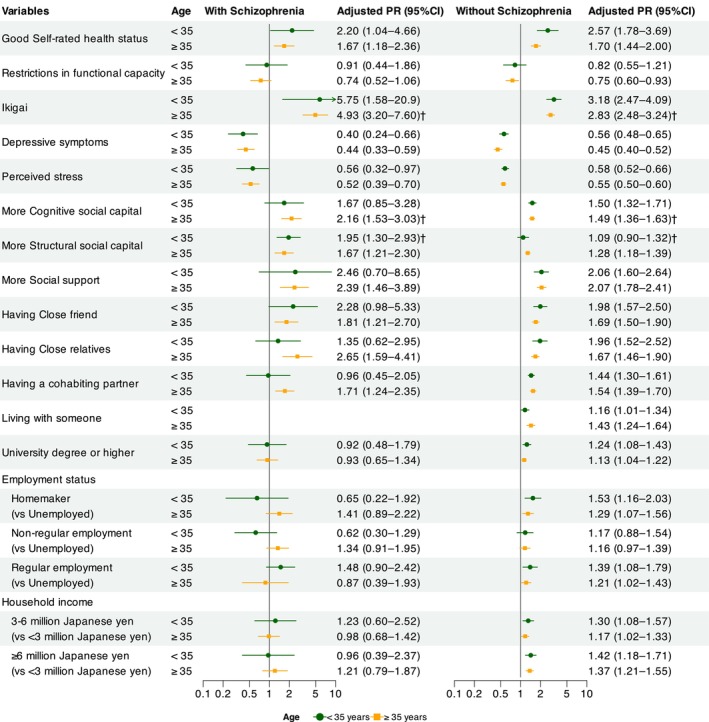
Sex‐adjusted prevalence ratios and 95% confidence intervals for happiness in participants with and without Schizophrenia, Stratified by Age. Adjusted PR, Sex‐adjusted prevalence ratio. ^†^ *p* < 0.05 for the difference in PRs (*P*_diff_) between participants with and without schizophrenia.

Figure 3 shows the PRs and 95% CIs for participants with and without schizophrenia, stratified by age. In younger age groups, structural social capital was found to be more strongly associated with happiness in participants with schizophrenia than in those without schizophrenia (with schizophrenia: PR = 1.95, 95% CI = 1.30–2.93 vs. without schizophrenia: PR = 1.09, 95% CI = 0.90–1.32, *P*_diff_ = 0.011). In the older age group, Ikigai (with schizophrenia: PR = 4.93, 95% CI = 3.20–7.60 vs. without schizophrenia: PR = 2.83, 95% CI = 2.48–3.24, *P*_diff_ = 0.016) and cognitive social capital (with schizophrenia: PR = 2.16, 95% CI = 1.53–3.03 vs. without schizophrenia: PR = 1.49, 95% CI = 1.36–1.63, *P*_diff_ = 0.040) were associated with a stronger sense of happiness in participants with schizophrenia than in those without schizophrenia. No significant differences were identified when comparing younger and older subgroups of participants with schizophrenia.

The results of the sensitivity analysis, which used an ordinal logistic regression model, were largely consistent with our primary findings, confirming the robustness of the main associations reported (Figure S1, Figure S2, and Figure S3). While the overall direction and significance of the main associations were upheld, some differences in the interaction terms were observed, as detailed in the Supporting Information.

## 4 Discussion

This study examined the characteristics of happiness in individuals with schizophrenia living in Japan. Our study findings revealed that SRHS, Ikigai, depressive symptoms, perceived stress, social capital (both cognitive and structural dimensions), social support, social relationships (close friends and relatives), and marital status were significantly associated with happiness, regardless of the presence of schizophrenia. Stratified analyses within the schizophrenia group by age and sex revealed that cognitive social capital and marital status were associated with happiness in a sex‐specific manner. In contrast, SRHS, Ikigai, depressive symptoms, perceived stress, structural social capital, social support, and social relationships were related to happiness, irrespective of age or sex. Furthermore, a comparison with participants without schizophrenia revealed that individuals with schizophrenia exhibited stronger associations between happiness and factors such as Ikigai, cognitive social capital, and structural social capital, with variations in these associations according to sex and age.

Individuals with schizophrenia reported lower levels of happiness than those without schizophrenia, which is consistent with previous studies in Western populations [5, 6, 7]. In participants with schizophrenia, SRHS, Ikigai, depressive symptoms, perceived stress, social support, close friends/relatives, and marital status were significantly associated with happiness; social factors relevant to their sense of happiness were mainly related to their interpersonal relationships. In particular, Ikigai and cognitive and structural social capital demonstrated stronger associations with happiness in individuals with schizophrenia than in those without. Other factors, such as functional capacity, residential status, educational background, employment status, and income, were not significantly associated. These results indicate that the factors associated with happiness among individuals with schizophrenia are predominantly related to good physical and mental health, as well as social and interpersonal connections. In contrast, factors not associated with happiness were primarily related to socioeconomic status.

Our findings are consistent with a previous study in American populations [6], which reported that lower perceived stress was associated with happiness, while educational background was not. That study also provides context for our finding on SRHS. The previous study analyzed health components separately using the MOS Study 36‐Item Short‐Form (SF‐36) and found a positive association between happiness and the mental health component (Spearman's *r* = 0.663, *p* < 0.001), but not the physical component (Spearman's *r* = 0.142). As our SRHS measure encompasses multiple aspects, including a mental component, our result is predominantly attributable to this association, suggesting that the subjective perception of mental health could be an important factor in the happiness of individuals with schizophrenia.

Our analyses demonstrated that the association between Ikigai and happiness was markedly stronger in individuals with schizophrenia than in those without. While previous research has documented a positive association between Ikigai and happiness in the general elderly Japanese population [37], studies examining this association specifically in individuals with schizophrenia are lacking. Although Ikigai shares conceptual similarities with happiness, it is distinguished by its temporal orientation: happiness primarily reflects present emotional states, whereas Ikigai encompasses future‐oriented purpose and meaning [38]. The heightened association between Ikigai and happiness suggests that future‐oriented perspectives may be particularly important for happiness in individuals with schizophrenia.

Similarly, both dimensions of social capital, cognitive (trust in others) and structural (participation in social activities), demonstrated significantly stronger associations with happiness in participants with schizophrenia than in those without schizophrenia. Although these components of social capital have been reported to correlate with happiness in local Japanese communities [10], their association with happiness has not been investigated in any population with schizophrenia. The present findings expand the existing literature by highlighting the potentially critical role of social connections in promoting happiness in individuals with schizophrenia.

Stratified analyses by sex and age were conducted to examine the differences in the associations between happiness and each variable (1) within the schizophrenia group and (2) between individuals with and without schizophrenia. In the sex‐stratified analysis comparing female participants with and without schizophrenia, no significant differences were observed in the associations based on the schizophrenia status. Conversely, among male participants with schizophrenia, cognitive social capital demonstrated a significantly stronger association with happiness than in those without. The sex variations in these associations may be partially explained by previous findings that women with schizophrenia tend to experience better social adaptation than men [39, 40].

Consistent with our hypothesis regarding sex differences in social factors, our study found that men with schizophrenia who had higher cognitive social capital exhibited greater subjective happiness than men without schizophrenia; this association was stronger in men than in women with schizophrenia. To our knowledge, the relationship between cognitive social capital and subjective happiness in individuals with schizophrenia has not been investigated extensively. Our findings suggest that cognitive social capital plays a crucial role in determining happiness in men with schizophrenia. However, negative symptoms associated with lower subjective well‐being in schizophrenia [9], have also been linked to impaired social functioning [41], including deficits in interpersonal skills and reduced participation in social activities. Such impairments in social functioning may influence cognitive social capital, suggesting that negative symptoms may confound the relationship between cognitive social capital and subjective happiness. This potential confounding effect may be particularly relevant for men, as previous studies have demonstrated that men with schizophrenia tend to exhibit more severe negative symptoms than do women [42, 43]. Future research should systematically examine schizophrenia‐specific symptoms as potential confounding variables, with particular attention paid to sex differences in the symptom severity and their impact on social capital and happiness.

Furthermore, in the comparison within the schizophrenia group, not only cognitive social capital but also having a cohabiting partner was significantly more strongly associated with happiness in men with schizophrenia than in women with schizophrenia. Our findings regarding the relationship between marital status and happiness in men with schizophrenia are consistent with those of previous research in the general Japanese population [44], which demonstrated that men's happiness was significantly associated with marital status (including singlehood, widowhood, and divorce). This finding suggests that marital status may be an important factor in determining happiness in different male populations.

Contrary to our hypothesis, which predicted that associations between mental factors and happiness would differ across age groups, an age‐stratified analysis of participants with schizophrenia demonstrated no significant differences in the association of factors with happiness between age groups. In an age‐stratified analysis comparing individuals with and without schizophrenia, structural social capital was more strongly associated with happiness in participants aged < 35 years than in those without schizophrenia. Among participants aged ≥ 35, cognitive social capital and Ikigai were more strongly associated with happiness in those with schizophrenia than in those without schizophrenia. No previous studies have examined these differences in the associations of structural and cognitive social capital and Ikigai with happiness in individuals with and without schizophrenia across different age groups. The results of this study indicate that while the characteristics of happiness‐related factors vary by the age group, depending on the presence or absence of schizophrenia, the relationship between the investigated factors and happiness among individuals with schizophrenia did not exhibit significant changes across different age groups. When examining the relationship between the investigated factors and happiness among individuals with schizophrenia, sex differences may be more important than age group differences.

This study had certain limitations that warrant consideration. First, the online survey methodology may have introduced a selection bias, potentially underrepresenting individuals with limited Internet access or digital literacy. This sampling limitation may have affected the generalizability of our findings. Second, we did not collect data on the positive and negative symptoms of schizophrenia, preventing us from controlling for clinical factors that may influence happiness and introduce confounding variables. Third, we did not account for the types of antipsychotic medications used by participants. Negative symptoms are a core part of schizophrenia spectrum pathology and can be secondary to other psychotic symptoms or caused by antipsychotic medication [45]. Second generation antipsychotics (SGAs) are often more effective than first generation antipsychotics (FGAs) in treating negative symptoms [46]. SGAs are generally associated with a lower risk of extrapyramidal symptoms that can cause secondary negative symptoms than FGAs [46]. Therefore, the differences in antipsychotics which participants received might have influenced our results. Fourth, the diagnosis of schizophrenia was based on self‐reports, which represent a significant limitation affecting both diagnostic validity and generalizability. While we implemented screening questions based on Diagnostic and Statistical Manual of Mental Disorders, Fifth Edition (DSM‐5) criteria and excluded other mental disorders to enhance accuracy, this approach may have introduced selection bias toward higher‐functioning individuals with sufficient insight to participate in online research. Our findings may therefore be most applicable to individuals with relatively stable schizophrenia, limiting generalizability to those with more severe presentations. Future research using clinically confirmed diagnoses would strengthen these findings. Fifth, the low eligibility rate in schizophrenia cases (223/5584 = 4.0%) raises additional concerns about sample representativeness. Combined with the self‐report diagnostic limitations discussed above, this might suggest our sample represents a highly selected subset, further limiting generalizability to the broader schizophrenia population. Sixth, participants received compensation through the Rakuten Insight platform, which may have influenced participation motivation and introduced selection bias toward individuals seeking monetary rewards. Seventh, happiness was measured using a single‐item, 4‐point Likert scale. As this was a secondary analysis of data originally collected for estimating schizophrenia prevalence, happiness was included as a supplementary variable rather than a primary outcome. While single‐item happiness measures are commonly used in large‐scale surveys, our 4‐point scale has limited resolution compared to validated multi‐item scales or the 0 to 10 scales recommended for measuring subjective well‐being [47]. This constraint may limit our ability to detect subtle variations in happiness levels and raises concerns about measurement reliability, as single‐item measures cannot assess internal consistency. Therefore, our findings should be interpreted as exploratory, requiring validation through purpose‐designed studies using established happiness scales. Eighth, we dichotomized the four‐category happiness outcome for our primary analysis. To confirm the validity of this approach, we performed a sensitivity analysis using ordinal logistic regression, which confirmed the robustness of our main findings. Although the significance of interaction terms differed between this and our primary model, this is an expected statistical divergence when comparing PR with odds ratios. We prioritized the PR‐based interactions for their direct epidemiological interpretability. Ninth, we performed multiple subgroup comparisons without statistical adjustment for multiplicity. This approach increases the risk of Type I errors (false positives), and therefore, findings from these stratified analyses should be interpreted with caution. As our analyses were exploratory, the aim was to identify potential associations that could inform future research rather than to perform formal confirmatory testing. These hypothesis‐generating findings require confirmation in subsequent studies specifically designed to test these effect modifications. Finally, the cross‐sectional design precludes causal inferences regarding the relationship between happiness and various physical, mental, and social factors. In addition, although our quantitative approach enables population‐level comparisons and enhances generalizability, it may not capture the full complexity of happiness experiences in individuals with schizophrenia. Future research could benefit from complementary qualitative investigations to provide deeper insights into individual experiences and potentially uncover aspects of happiness that are not readily quantifiable. Such mixed‐method approaches are particularly valuable for informing community‐based service development and delivery.

In conclusion, happiness among individuals with schizophrenia is associated with good physical and mental health as well as social and interpersonal connections. Notably, differences in the social factors were observed between individuals with and without schizophrenia. Additionally, within the group of individuals with schizophrenia, sex differences were found in social capital and marital status, whereas comparisons based on the presence or absence of schizophrenia revealed age group differences in Ikigai and social capital. These findings suggest that interventions aimed at enhancing the happiness of individuals with schizophrenia should consider individual characteristics, particularly sex differences, and focus on social factors such as interpersonal and community connections.

## Author Contributions

Conceptualization, J.H., M.M., T.K., and A.O.; methodology, M.M., T.K., and A.O.; software, J.H. and M.M.; validation, J.H., M.M., and A.O.; formal analysis, J.H. and M.M.; investigation, A.O.; resources, A.O.; data curation, J.H., M.M., and A.O.; writing – original draft preparation, J.H. and M.M.; writing – review and editing, M.M., Y.H., K.S., T.K., S.T., N.I., and A.O.; visualization, J.H. and M.M.; supervision, A.O.; project administration, A.O.; funding acquisition, A.O. All authors have read and agreed to the published version of the manuscript.

## Ethics Statement

The study was conducted in accordance with the Declaration of Helsinki and approved by the institutional review board of the Fujita Health University (no. HM21‐408 and HM23‐253).

## Consent

Informed consent was obtained from all study participants via the Internet. Participants were presented with detailed information regarding study purpose, data use, management responsibilities, and their rights, and confirmed their understanding and consent through a mandatory electronic checkbox.

## Conflicts of Interest

The authors declare no conflicts of interest.

## Supporting information


**Table S1:** Descriptive statistics of demographic characteristics and key study variables among men.
**Table S2:** Descriptive statistics of demographic characteristics and key study variables among women.
**Table S3:** Descriptive statistics of demographic characteristics and key study variables stratified by age in participants with schizophrenia.
**Table S4:** Descriptive statistics of demographic characteristics and key study variables stratified by age in participants without schizophrenia.
**Table S5:** Descriptive statistics of demographic characteristics and key study variables stratified by sex in participants with schizophrenia.
**Table S6:** Descriptive statistics of demographic characteristics and key study variables stratified by sex in participants without schizophrenia.

**Figure S1:** Sex‐ and Age‐adjusted Odds Ratios and 95% Confidence Intervals for Happiness in Participants with and without Schizophrenia.
**Figure S2:** Age‐adjusted Odds Ratios and 95% Confidence Intervals for Happiness in Participants with and without Schizophrenia, Stratified by Sex.
**Figure S3:** Sex‐adjusted Odds Ratios and 95% Confidence Intervals for Happiness in Participants with and without Schizophrenia, Stratified by Age.

## Data Availability

The raw data supporting the findings of this study are not publicly available because data‐sharing approval was not obtained from the institutional review board of the Fujita Health University. However, while the raw data are not available, detailed descriptive statistics are provided as (Tables S1–S6). These tables present a comparison of demographic and key study variables between participants with and without schizophrenia. The data are further stratified by age group and sex. The survey items, presented in their original Japanese, are also described in these tables. Inquiries regarding the dataset or requests for additional descriptive statistics may be directed to the corresponding author.
