# Supplementary material for: Physical, Mental, and Social Characteristics Associated With Happiness in Individuals With Schizophrenia in Japan: A Cross‐Sectional Study
Source: Neuropsychopharmacol Rep. 2025 Sep 4;45(3):e70045. doi: 10.1002/npr2.70045 (PMC12409470; doi:10.1002/npr2.70045)
Supplement: Supplementary file 1 — Table S1: Descriptive statistics of demographic characteristics and key study variables among men. Table S2: Descriptive statistics of demographic characteristics and key study variables among women. Table S3: Descriptive statistics of demographic characteristics and key study variables stratified by age in participants with schizophrenia. Table S4: Descriptive statistics of demographic characteristics and key study variables stratified by age in participants without schizophrenia. Table S5: Descriptive statistics of demographic characteristics and key study variables stratified by sex in participants with schizophrenia. Table S6: Descriptive statistics of demographic characteristics and key study variables stratified by sex in participants without schizophrenia. [file NPR2-45-e70045-s002.pdf]

Table S1 Descriptive statistics of demographic characteristics and key study variables among men

| 質問項目[1] | 統合失調症なし N = 801[2] | 統合失調症あり N = 115[2] |
|---|---|---|
| **年齢** | 49 (38, 58) | 48 (42, 54) |
| **性別** | | |
| 男性 | 801 (100%) | 115 (100%) |
| 女性 | 0 (0%) | 0 (0%) |
| **あなたの現在の身長および体重をお答えください。（半角数字でご記入ください）※小数点以下は四捨五入してください。身長（cm）** | 171.0 (167.0, 175.0) | 171.0 (166.0, 174.0) |
| **あなたの現在の身長および体重をお答えください。（半角数字でご記入ください）※小数点以下は四捨五入してください。体重（kg）** | 68 (60, 75) | 75 (65, 88) |
| **現在の結婚状況についてお聞きします。1つだけ選択してください。** | | |
| 結婚・再婚・内縁（いずれも別居は除く） | 463 (58%) | 21 (18%) |
| 離婚 | 37 (4.6%) | 6 (5.2%) |
| 別居 | 6 (0.7%) | 0 (0%) |
| 死別 | 2 (0.2%) | 0 (0%) |
| 未婚 | 286 (36%) | 88 (77%) |
| その他 | 7 (0.9%) | 0 (0%) |
| **現在、どなたと一緒にお住まいですか。同居しているすべての人を選択してください。（いくつでも）（同居・配偶者）** | | |
| なし | 345 (43%) | 94 (82%) |
| あり | 456 (57%) | 21 (18%) |
| **現在、どなたと一緒にお住まいですか。同居しているすべての人を選択してください。（いくつでも）（同居・子供）** | | |
| なし | 544 (68%) | 100 (87%) |
| あり | 257 (32%) | 15 (13%) |
| **現在、どなたと一緒にお住まいですか。同居しているすべての人を選択してください。（いくつでも）（同居・親）** | | |
| なし | 607 (76%) | 42 (37%) |
| あり | 194 (24%) | 73 (63%) |
| **現在、どなたと一緒にお住まいですか。同居しているすべての人を選択してください。（いくつでも）（同居・その他）** | | |
| なし | 751 (94%) | 88 (77%) |
| あり | 50 (6.2%) | 27 (23%) |

[1] Note: Items shown in this table were presented in Japanese in the original survey.

[2] Median (Q1, Q3); n (%)

| 質問項目[1] | 統合失調症なし N = 801[2] | 統合失調症あり N = 115[2] |
|---|---|---|
| **現在、どなたと一緒にお住まいですか。同居しているすべての人を選択してください。（いくつでも）（同居・独り暮らし）** | | |
| なし | 624 (78%) | 93 (81%) |
| あり | 177 (22%) | 22 (19%) |
| **現在の同居人数は何人ですか。（半角数字でご記入ください）※あなたは含めません。** | | |
| 1 | 163 (26%) | 21 (23%) |
| 2 | 178 (29%) | 39 (42%) |
| 3 | 162 (26%) | 20 (22%) |
| 4 | 83 (13%) | 9 (9.7%) |
| 5 | 25 (4.0%) | 3 (3.2%) |
| 6 | 10 (1.6%) | 0 (0%) |
| 7 | 1 (0.2%) | 0 (0%) |
| 8 | 2 (0.3%) | 1 (1.1%) |
| 回答なし | 177 | 22 |
| **現在の同居人数は何人ですか。（半角数字でご記入ください）※あなたは含めません。（年齢14歳未満の同居人数）** | | |
| 0 | 108 (42%) | 8 (53%) |
| 1 | 81 (32%) | 3 (20%) |
| 2 | 59 (23%) | 4 (27%) |
| 3 | 8 (3.1%) | 0 (0%) |
| 4 | 1 (0.4%) | 0 (0%) |
| 回答なし | 544 | 100 |
| **全体的にみて、あなたの過去1か月間の健康状態はいかがでしたか。** | | |
| 最高に良い | 92 (11%) | 5 (4.3%) |
| やや良い | 176 (22%) | 24 (21%) |
| 良い | 397 (50%) | 35 (30%) |
| あまり良くない | 116 (14%) | 42 (37%) |
| 良くない | 20 (2.5%) | 9 (7.8%) |
| **現在の日常生活の状態はいかがですか。最も当てはまると思われるものを選んでください。** | | |
| 身体に特に障害はない | 742 (93%) | 70 (61%) |
| ＜身体に何らかの障害はあるが、日常生活はほぼ自分で出来、独力で外出する＞交通機関などを利用して外出する | 48 (6.0%) | 15 (13%) |
| ＜身体に何らかの障害はあるが、日常生活はほぼ自分で出来、独力で外出する＞隣近所にのみ出かける | 2 (0.2%) | 22 (19%) |

[1] Note: Items shown in this table were presented in Japanese in the original survey.

[2] Median (Q1, Q3); n (%)

| **質問項目**[1] | **統合失調症なし** N = 801[2] | **統合失調症あり** N = 115[2] |
|---|---|---|
| ＜屋内での生活はおおむね自分で出来るが、介助なしには外出しない＞介助をしてもらって外出し、日中はほとんどベッドから離れて生活する | 1 (0.1%) | 2 (1.7%) |
| ＜屋内での生活はおおむね自分で出来るが、介助なしには外出しない＞外出の頻度が少なく、日中も寝たり起きたりの生活をしている | 1 (0.1%) | 4 (3.5%) |
| ＜屋内での生活は何らかの介助を必要とし、日中もベッド上での生活が主であるが、座位を保つことができる＞車椅子に自分で乗り、食事、排泄はベッドから離れて行う | 1 (0.1%) | 1 (0.9%) |
| ＜屋内での生活は何らかの介助を必要とし、日中もベッド上での生活が主であるが、座位を保つことができる＞介助により車椅子に乗る | 0 (0%) | 1 (0.9%) |
| ＜1日中ベッド上で過ごし、排泄、食事、着替えの時に介助が要る＞自力で寝返りを打つ | 0 (0%) | 0 (0%) |
| ＜1日中ベッド上で過ごし、排泄、食事、着替えの時に介助が要る＞自力では寝返りも打たない | 6 (0.7%) | 0 (0%) |
| **昨年1年間の「身体の動かし方」についておたずねします。昨年1年間のうち、通常の時期の1日の時間の内訳を教えてください。通勤、仕事、家事などの時間をすべて含めてお答えください。余暇は含めません。: 1.座っている時間** | | |
| なかった | 23 (2.9%) | 0 (0%) |
| 1時間未満 | 20 (2.5%) | 9 (7.8%) |
| 1時間以上3時間未満 | 127 (16%) | 25 (22%) |
| 3時間以上5時間未満 | 208 (26%) | 25 (22%) |
| 5時間以上7時間未満 | 200 (25%) | 25 (22%) |
| 7時間以上9時間未満 | 105 (13%) | 10 (8.7%) |
| 9時間以上11時間未満 | 50 (6.2%) | 7 (6.1%) |
| 11時間以上 | 68 (8.5%) | 14 (12%) |
| **昨年1年間の「身体の動かし方」についておたずねします。昨年1年間のうち、通常の時期の1日の時間の内訳を教えてください。通勤、仕事、家事などの時間をすべて含めてお答えください。余暇は含めません。: 2.立っている時間** | | |
| なかった | 29 (3.6%) | 6 (5.2%) |
| 1時間未満 | 157 (20%) | 37 (32%) |
| 1時間以上3時間未満 | 304 (38%) | 43 (37%) |
| 3時間以上5時間未満 | 169 (21%) | 13 (11%) |
| 5時間以上7時間未満 | 76 (9.5%) | 8 (7.0%) |
| 7時間以上9時間未満 | 38 (4.7%) | 4 (3.5%) |
| 9時間以上11時間未満 | 17 (2.1%) | 3 (2.6%) |
| 11時間以上 | 11 (1.4%) | 1 (0.9%) |
| **昨年1年間の「身体の動かし方」についておたずねします。昨年1年間のうち、通常の時期の1日の時間の内訳を教えてください。通勤、仕事、家事などの時間をすべて含めてお答えください。余暇は含めません。: 3.歩いている時間** | | |

[1] Note: Items shown in this table were presented in Japanese in the original survey.

[2] Median (Q1, Q3); n (%)

| 質問項目[1] | 統合失調症なし<br>N = 801[2] | 統合失調症あり<br>N = 115[2] |
|---|---|---|
| なかった | 32 (4.0%) | 7 (6.1%) |
| 1時間未満 | 410 (51%) | 59 (51%) |
| 1時間以上3時間未満 | 286 (36%) | 44 (38%) |
| 3時間以上5時間未満 | 51 (6.4%) | 3 (2.6%) |
| 5時間以上7時間未満 | 8 (1.0%) | 0 (0%) |
| 7時間以上9時間未満 | 5 (0.6%) | 1 (0.9%) |
| 9時間以上11時間未満 | 5 (0.6%) | 1 (0.9%) |
| 11時間以上 | 4 (0.5%) | 0 (0%) |
| **昨年1年間の「身体の動かし方」についておたずねします。昨年1年間のうち、通常の時期の1日の時間の内訳を教えてください。通勤、仕事、家事などの時間をすべて含めてお答えください。余暇は含めません。: 4.力のいる作業をしている時間** | | |
| なかった | 276 (34%) | 61 (53%) |
| 1時間未満 | 371 (46%) | 36 (31%) |
| 1時間以上3時間未満 | 101 (13%) | 10 (8.7%) |
| 3時間以上5時間未満 | 33 (4.1%) | 4 (3.5%) |
| 5時間以上7時間未満 | 12 (1.5%) | 0 (0%) |
| 7時間以上9時間未満 | 5 (0.6%) | 2 (1.7%) |
| 9時間以上11時間未満 | 2 (0.2%) | 1 (0.9%) |
| 11時間以上 | 1 (0.1%) | 1 (0.9%) |
| **あなたの現在の状況について、以下の質問にお答えください。: 1.必要な時に、あなたの話を聞いてくれる人がいますか** | | |
| ほとんどいない | 129 (16%) | 19 (17%) |
| たまにいる | 132 (16%) | 23 (20%) |
| ときどきいる | 185 (23%) | 27 (23%) |
| よくいる | 174 (22%) | 19 (17%) |
| いつでもいる | 181 (23%) | 27 (23%) |
| **あなたの現在の状況について、以下の質問にお答えください。: 2.なにか困ったことがあった時、よいアドバイスをくれる人がいますか** | | |
| ほとんどいない | 164 (20%) | 24 (21%) |
| たまにいる | 167 (21%) | 25 (22%) |
| ときどきいる | 209 (26%) | 23 (20%) |
| よくいる | 140 (17%) | 22 (19%) |

[1] Note: Items shown in this table were presented in Japanese in the original survey.

[2] Median (Q1, Q3); n (%)

| 質問項目[1] | 統合失調症なし<br>N = 801[2] | 統合失調症あり<br>N = 115[2] |
|---|---|---|
| いつでもいる | 121 (15%) | 21 (18%) |
| **あなたの現在の状況について、以下の質問にお答えください。: 3.あなたを心配したり、あなたに愛情をかけてくれる人はいますか** | | |
| ほとんどいない | 111 (14%) | 17 (15%) |
| たまにいる | 153 (19%) | 20 (17%) |
| ときどきいる | 190 (24%) | 31 (27%) |
| よくいる | 156 (19%) | 19 (17%) |
| いつでもいる | 191 (24%) | 28 (24%) |
| **あなたの現在の状況について、以下の質問にお答えください。: 4.日常の家事をしたり、手伝ってくれる人はいますか** | | |
| ほとんどいない | 156 (19%) | 20 (17%) |
| たまにいる | 101 (13%) | 19 (17%) |
| ときどきいる | 152 (19%) | 12 (10%) |
| よくいる | 164 (20%) | 28 (24%) |
| いつでもいる | 228 (28%) | 36 (31%) |
| **あなたの現在の状況について、以下の質問にお答えください。: 5.あなたに情緒的な支えを与えてくれるような人、（たとえば、あなたの直面する問題について相談できる人、難しい判断が必要な時に助けてくれる人）はいますか** | | |
| ほとんどいない | 180 (22%) | 24 (21%) |
| たまにいる | 116 (14%) | 23 (20%) |
| ときどきいる | 183 (23%) | 23 (20%) |
| よくいる | 155 (19%) | 20 (17%) |
| いつでもいる | 167 (21%) | 25 (22%) |
| **あなたの現在の状況について、以下の質問にお答えください。: 6.必要な時にいつでも連絡がとれる、親しくて、信頼・信用できる人はいますか** | | |
| ほとんどいない | 149 (19%) | 23 (20%) |
| たまにいる | 133 (17%) | 22 (19%) |
| ときどきいる | 193 (24%) | 25 (22%) |
| よくいる | 148 (18%) | 19 (17%) |
| いつでもいる | 178 (22%) | 26 (23%) |
| **あなたの現在の状況について、以下の質問にお答えください。: 1.気軽に個人的な相談ができる親しい友人は何人いますか** | | |
| 0人 | 282 (35%) | 56 (49%) |

[1] Note: Items shown in this table were presented in Japanese in the original survey.

[2] Median (Q1, Q3); n (%)

| 質問項目[1] | 統合失調症なし N = 801[2] | 統合失調症あり N = 115[2] |
|---|---|---|
| 1人 | 166 (21%) | 25 (22%) |
| 2人 | 159 (20%) | 17 (15%) |
| 3人以上 | 194 (24%) | 17 (15%) |
| **あなたの現在の状況について、以下の質問にお答えください。: 2.気軽に個人的な相談ができる親類は何人いますか** | | |
| 0人 | 221 (28%) | 36 (31%) |
| 1人 | 210 (26%) | 32 (28%) |
| 2人 | 174 (22%) | 24 (21%) |
| 3人以上 | 196 (24%) | 23 (20%) |
| **地域組織、自助集団、チャリティー、ボランティアグループや、宗教団体などの集まりにどれくらいの頻度で参加していますか。** | | |
| 全く／ほとんど参加しない | 678 (85%) | 99 (86%) |
| 時々参加する | 87 (11%) | 5 (4.3%) |
| 週に1回未満 | 16 (2.0%) | 4 (3.5%) |
| 週に1回以上 | 20 (2.5%) | 7 (6.1%) |
| **あなたは、生きがいがあると感じていますか。** | | |
| 非常にある | 101 (13%) | 14 (12%) |
| ある | 399 (50%) | 35 (30%) |
| あまりない | 219 (27%) | 46 (40%) |
| 全くない | 82 (10%) | 20 (17%) |
| **あなたはご自分がどれくらい幸せだと感じていますか。** | | |
| 大変幸せ | 100 (12%) | 10 (8.7%) |
| 幸せ | 394 (49%) | 32 (28%) |
| どちらとも言えない | 237 (30%) | 43 (37%) |
| 幸せでない | 70 (8.7%) | 30 (26%) |
| **最近1週間の体や心の状態について、お聞きします。それぞれ最もあてはまる選択肢1つ選択してください。: 1.食べたくない。食欲がおちた** | | |
| 全く／ほとんどなかった | 668 (83%) | 87 (76%) |
| たまにあった（1〜2日） | 90 (11%) | 19 (17%) |
| しばしばあった（3〜4日） | 27 (3.4%) | 5 (4.3%) |
| いつもあった（ほぼ毎日） | 16 (2.0%) | 4 (3.5%) |

[1] Note: Items shown in this table were presented in Japanese in the original survey.

[2] Median (Q1, Q3); n (%)

| 質問項目[1] | 統合失調症なし N = 801[2] | 統合失調症あり N = 115[2] |
|---|---|---|
| **最近1週間の体や心の状態について、お聞きします。それぞれ最もあてはまる選択肢1つ選択してください。: 2.ゆううつだ** | | |
| 全く／ほとんどなかった | 499 (62%) | 29 (25%) |
| たまにあった（1〜2日） | 203 (25%) | 43 (37%) |
| しばしばあった（3〜4日） | 66 (8.2%) | 19 (17%) |
| いつもあった（ほぼ毎日） | 33 (4.1%) | 24 (21%) |
| **最近1週間の体や心の状態について、お聞きします。それぞれ最もあてはまる選択肢1つ選択してください。: 3.何をするのも面倒だ** | | |
| 全く／ほとんどなかった | 445 (56%) | 25 (22%) |
| たまにあった（1〜2日） | 253 (32%) | 45 (39%) |
| しばしばあった（3〜4日） | 65 (8.1%) | 18 (16%) |
| いつもあった（ほぼ毎日） | 38 (4.7%) | 27 (23%) |
| **最近1週間の体や心の状態について、お聞きします。それぞれ最もあてはまる選択肢1つ選択してください。: 4.なかなか眠れない** | | |
| 全く／ほとんどなかった | 558 (70%) | 46 (40%) |
| たまにあった（1〜2日） | 180 (22%) | 28 (24%) |
| しばしばあった（3〜4日） | 45 (5.6%) | 15 (13%) |
| いつもあった（ほぼ毎日） | 18 (2.2%) | 26 (23%) |
| **最近1週間の体や心の状態について、お聞きします。それぞれ最もあてはまる選択肢1つ選択してください。: 5.生活について満足して過ごせる** | | |
| 全く／ほとんどなかった | 218 (27%) | 34 (30%) |
| たまにあった（1〜2日） | 162 (20%) | 35 (30%) |
| しばしばあった（3〜4日） | 227 (28%) | 23 (20%) |
| いつもあった（ほぼ毎日） | 194 (24%) | 23 (20%) |
| **最近1週間の体や心の状態について、お聞きします。それぞれ最もあてはまる選択肢1つ選択してください。: 6.一人ぼっちでさびしい** | | |
| 全く／ほとんどなかった | 633 (79%) | 64 (56%) |
| たまにあった（1〜2日） | 107 (13%) | 31 (27%) |
| しばしばあった（3〜4日） | 28 (3.5%) | 12 (10%) |
| いつもあった（ほぼ毎日） | 33 (4.1%) | 8 (7.0%) |
| **最近1週間の体や心の状態について、お聞きします。それぞれ最もあてはまる選択肢1つ選択してください。: 7.皆がよそよそしいと思う** | | |
| 全く／ほとんどなかった | 638 (80%) | 66 (57%) |

[1] Note: Items shown in this table were presented in Japanese in the original survey.

[2] Median (Q1, Q3); n (%)

| 質問項目[1] | 統合失調症なし<br>N = 801[2] | 統合失調症あり<br>N = 115[2] |
|---|---|---|
| たまにあった（1〜2日） | 122 (15%) | 27 (23%) |
| しばしばあった（3〜4日） | 26 (3.2%) | 14 (12%) |
| いつもあった（ほぼ毎日） | 15 (1.9%) | 8 (7.0%) |
| **最近1週間の体や心の状態について、お聞きします。それぞれ最もあてはまる選択肢1つ選択してください。: 8.毎日が楽しい** | | |
| 全く／ほとんどなかった | 189 (24%) | 41 (36%) |
| たまにあった（1〜2日） | 270 (34%) | 45 (39%) |
| しばしばあった（3〜4日） | 207 (26%) | 18 (16%) |
| いつもあった（ほぼ毎日） | 135 (17%) | 11 (9.6%) |
| **最近1週間の体や心の状態について、お聞きします。それぞれ最もあてはまる選択肢1つ選択してください。: 9.悲しいと感じる** | | |
| 全く／ほとんどなかった | 527 (66%) | 43 (37%) |
| たまにあった（1〜2日） | 207 (26%) | 41 (36%) |
| しばしばあった（3〜4日） | 46 (5.7%) | 20 (17%) |
| いつもあった（ほぼ毎日） | 21 (2.6%) | 11 (9.6%) |
| **最近1週間の体や心の状態について、お聞きします。それぞれ最もあてはまる選択肢1つ選択してください。: 10.皆が自分を嫌っていると感じる** | | |
| 全く／ほとんどなかった | 636 (79%) | 55 (48%) |
| たまにあった（1〜2日） | 124 (15%) | 29 (25%) |
| しばしばあった（3〜4日） | 28 (3.5%) | 14 (12%) |
| いつもあった（ほぼ毎日） | 13 (1.6%) | 17 (15%) |
| **最近1週間の体や心の状態について、お聞きします。それぞれ最もあてはまる選択肢1つ選択してください。: 11.仕事が手につかない** | | |
| 全く／ほとんどなかった | 613 (77%) | 54 (47%) |
| たまにあった（1〜2日） | 149 (19%) | 31 (27%) |
| しばしばあった（3〜4日） | 27 (3.4%) | 10 (8.7%) |
| いつもあった（ほぼ毎日） | 12 (1.5%) | 20 (17%) |
| **あなたの気持ち、考えなどについてお伺いします。: 1.過去1か月、「人生での大切な事が自分の思うようにならない」と感じましたか** | | |
| 全くない | 222 (28%) | 10 (8.7%) |
| ほとんどない | 244 (30%) | 23 (20%) |
| ときどき | 251 (31%) | 37 (32%) |

[1] Note: Items shown in this table were presented in Japanese in the original survey.

[2] Median (Q1, Q3); n (%)

| 質問項目[1] | 統合失調症なし<br>N = 801[2] | 統合失調症あり<br>N = 115[2] |
|---|---|---|
| 頻繁に | 40 (5.0%) | 22 (19%) |
| とても頻繁に | 44 (5.5%) | 23 (20%) |
| **あなたの気持ち、考えなどについてお伺いします。: 2.過去1か月、「自分の問題を解決する能力に自信がある」と感じましたか** | | |
| 全くない | 132 (16%) | 31 (27%) |
| ほとんどない | 187 (23%) | 37 (32%) |
| ときどき | 324 (40%) | 38 (33%) |
| 頻繁に | 127 (16%) | 8 (7.0%) |
| とても頻繁に | 31 (3.9%) | 1 (0.9%) |
| **あなたの気持ち、考えなどについてお伺いします。: 3.過去1か月、「思うように物事がいっている」と感じましたか** | | |
| 全くない | 127 (16%) | 26 (23%) |
| ほとんどない | 176 (22%) | 43 (37%) |
| ときどき | 377 (47%) | 35 (30%) |
| 頻繁に | 95 (12%) | 9 (7.8%) |
| とても頻繁に | 26 (3.2%) | 2 (1.7%) |
| **あなたの気持ち、考えなどについてお伺いします。: 4.過去1か月、「多くの困難が山積みで自分の手に負えない」と感じましたか** | | |
| 全くない | 242 (30%) | 13 (11%) |
| ほとんどない | 285 (36%) | 35 (30%) |
| ときどき | 199 (25%) | 36 (31%) |
| 頻繁に | 51 (6.4%) | 16 (14%) |
| とても頻繁に | 24 (3.0%) | 15 (13%) |
| **あなたの周りの状況について、以下の質問にお答えください。: 1.一般的に、人は信用できると思いますか** | | |
| 全く思わない | 87 (11%) | 19 (17%) |
| あまり思わない | 312 (39%) | 55 (48%) |
| 思う | 367 (46%) | 38 (33%) |
| 非常によく思う | 35 (4.4%) | 3 (2.6%) |
| **あなたの周りの状況について、以下の質問にお答えください。: 2.多くの人は隙さえあれば、他の人を利用しようとするものだと思いますか** | | |
| 全く思わない | 68 (8.5%) | 8 (7.0%) |

[1] Note: Items shown in this table were presented in Japanese in the original survey.

[2] Median (Q1, Q3); n (%)

| 質問項目[1] | 統合失調症なし<br>N = 801[2] | 統合失調症あり<br>N = 115[2] |
|---|---|---|
| あまり思わない | 392 (49%) | 48 (42%) |
| 思う | 297 (37%) | 46 (40%) |
| 非常によく思う | 44 (5.5%) | 13 (11%) |
| **あなたの周りの状況について、以下の質問にお答えください。: 3.多くの場合、人は他の人の役に立とうとすると思いますか** | | |
| 全く思わない | 69 (8.6%) | 11 (9.6%) |
| あまり思わない | 347 (43%) | 50 (43%) |
| 思う | 364 (45%) | 48 (42%) |
| 非常によく思う | 21 (2.6%) | 6 (5.2%) |
| **学校教育はどのくらいまで受けられましたか。1つだけ選択してください。** | | |
| 中学校 | 21 (2.6%) | 7 (6.1%) |
| 高校 | 192 (24%) | 43 (37%) |
| 短大卒・専門学校・4年制大学中退 | 102 (13%) | 17 (15%) |
| 大学以上 | 479 (60%) | 47 (41%) |
| その他 | 7 (0.9%) | 1 (0.9%) |
| **現在の従事している職業は何ですか。** | | |
| 無職 | 119 (15%) | 58 (50%) |
| 主婦（主夫） | 5 (0.6%) | 3 (2.6%) |
| 専門的・技術的職業従事者 | 140 (17%) | 5 (4.3%) |
| 管理的職業従事者 | 95 (12%) | 3 (2.6%) |
| 事務従事者 | 112 (14%) | 7 (6.1%) |
| 販売従事者 | 34 (4.2%) | 1 (0.9%) |
| サービス職業従事者 | 72 (9.0%) | 5 (4.3%) |
| 保安職業従事者 | 12 (1.5%) | 0 (0%) |
| 農業漁業作業者 | 5 (0.6%) | 2 (1.7%) |
| 運輸・通信従事者 | 32 (4.0%) | 2 (1.7%) |
| 生産工程・労務作業者 | 56 (7.0%) | 10 (8.7%) |
| その他の職業 | 119 (15%) | 19 (17%) |
| **現在従事されているお仕事の雇用形態は何ですか。** | | |
| 正社員・職員 | 489 (72%) | 14 (26%) |
| 契約社員・職員 | 44 (6.5%) | 4 (7.4%) |

[1] Note: Items shown in this table were presented in Japanese in the original survey.

[2] Median (Q1, Q3); n (%)

| 質問項目[1] | 統合失調症なし<br>N = 801[2] | 統合失調症あり<br>N = 115[2] |
|---|---|---|
| 派遣社員・職員 | 15 (2.2%) | 0 (0%) |
| パート・アルバイト | 44 (6.5%) | 26 (48%) |
| 自営・経営者 | 85 (13%) | 10 (19%) |
| 回答なし | 124 | 61 |
| **現在の世帯年収（税込み）はどのくらいですか。** | | |
| 0〜299万円 | 148 (18%) | 61 (53%) |
| 300〜599万円 | 280 (35%) | 40 (35%) |
| 600〜899万円 | 197 (25%) | 12 (10%) |
| 900〜1,199万円 | 104 (13%) | 1 (0.9%) |
| 1,200〜1,499万円 | 37 (4.6%) | 0 (0%) |
| 1,500万円以上 | 35 (4.4%) | 1 (0.9%) |

[1] Note: Items shown in this table were presented in Japanese in the original survey.

[2] Median (Q1, Q3); n (%)

**Table S2 Descriptive statistics of demographic characteristics and key study variables among women**

| 質問項目[1] | 統合失調症なし N = 975[2] | 統合失調症あり N = 108[2] |
|---|---|---|
| **年齢** | 41 (31, 51) | 45 (36, 50) |
| **性別** | | |
| 男性 | 0 (0%) | 0 (0%) |
| 女性 | 975 (100%) | 108 (100%) |
| **あなたの現在の身長および体重をお答えください。（半角数字でご記入ください）※小数点以下は四捨五入してください。身長（cm）** | 158.0 (154.0, 162.0) | 158.0 (155.0, 163.0) |
| **あなたの現在の身長および体重をお答えください。（半角数字でご記入ください）※小数点以下は四捨五入してください。体重（kg）** | 51 (47, 56) | 60 (51, 70) |
| **現在の結婚状況についてお聞きします。1つだけ選択してください。** | | |
| 結婚・再婚・内縁（いずれも別居は除く） | 519 (53%) | 41 (38%) |
| 離婚 | 67 (6.9%) | 14 (13%) |
| 別居 | 4 (0.4%) | 3 (2.8%) |
| 死別 | 10 (1.0%) | 2 (1.9%) |
| 未婚 | 371 (38%) | 48 (44%) |
| その他 | 4 (0.4%) | 0 (0%) |
| **現在、どなたと一緒にお住まいですか。同居しているすべての人を選択してください。（いくつでも）（同居・配偶者）** | | |
| なし | 463 (47%) | 67 (62%) |
| あり | 512 (53%) | 41 (38%) |
| **現在、どなたと一緒にお住まいですか。同居しているすべての人を選択してください。（いくつでも）（同居・子供）** | | |
| なし | 627 (64%) | 76 (70%) |
| あり | 348 (36%) | 32 (30%) |
| **現在、どなたと一緒にお住まいですか。同居しているすべての人を選択してください。（いくつでも）（同居・親）** | | |
| なし | 746 (77%) | 58 (54%) |
| あり | 229 (23%) | 50 (46%) |
| **現在、どなたと一緒にお住まいですか。同居しているすべての人を選択してください。（いくつでも）（同居・その他）** | | |
| なし | 884 (91%) | 91 (84%) |
| あり | 91 (9.3%) | 17 (16%) |

[1] Note: Items shown in this table were presented in Japanese in the original survey.

[2] Median (Q1, Q3); n (%)

| 質問項目[1] | 統合失調症なし<br>N = 975[2] | 統合失調症あり<br>N = 108[2] |
|---|---|---|
| **現在、どなたと一緒にお住まいですか。同居しているすべての人を選択してください。（いくつでも）（同居・独り暮らし）** | | |
| なし | 784 (80%) | 97 (90%) |
| あり | 191 (20%) | 11 (10%) |
| **現在の同居人数は何人ですか。（半角数字でご記入ください）※あなたは含めません。** | | |
| 1 | 200 (26%) | 31 (32%) |
| 2 | 247 (32%) | 34 (35%) |
| 3 | 201 (26%) | 23 (24%) |
| 4 | 105 (13%) | 6 (6.2%) |
| 5 | 23 (2.9%) | 2 (2.1%) |
| 6 | 7 (0.9%) | 1 (1.0%) |
| 7 | 1 (0.1%) | 0 (0%) |
| 回答なし | 191 | 11 |
| **現在の同居人数は何人ですか。（半角数字でご記入ください）※あなたは含めません。（年齢14歳未満の同居人数）** | | |
| 0 | 108 (31%) | 12 (38%) |
| 1 | 141 (41%) | 14 (44%) |
| 2 | 77 (22%) | 6 (19%) |
| 3 | 21 (6.0%) | 0 (0%) |
| 4 | 1 (0.3%) | 0 (0%) |
| 回答なし | 627 | 76 |
| **全体的にみて、あなたの過去1か月間の健康状態はいかがでしたか。** | | |
| 最高に良い | 103 (11%) | 1 (0.9%) |
| やや良い | 256 (26%) | 9 (8.3%) |
| 良い | 474 (49%) | 41 (38%) |
| あまり良くない | 127 (13%) | 42 (39%) |
| 良くない | 15 (1.5%) | 15 (14%) |
| **現在の日常生活の状態はいかがですか。最も当てはまると思われるものを選んでください。** | | |
| 身体に特に障害はない | 930 (95%) | 64 (59%) |
| ＜身体に何らかの障害はあるが、日常生活はほぼ自分で出来、独力で外出する＞交通機関などを利用して外出する | 29 (3.0%) | 21 (19%) |
| ＜身体に何らかの障害はあるが、日常生活はほぼ自分で出来、独力で外出する＞隣近所にのみ出かける | 4 (0.4%) | 17 (16%) |

[1] Note: Items shown in this table were presented in Japanese in the original survey.

[2] Median (Q1, Q3); n (%)

| 質問項目[1] | 統合失調症なし N = 975[2] | 統合失調症あり N = 108[2] |
|---|---|---|
| ＜屋内での生活はおおむね自分で出来るが、介助なしには外出しない＞介助をしてもらって外出し、日中はほとんどベッドから離れて生活する | 1 (0.1%) | 3 (2.8%) |
| ＜屋内での生活はおおむね自分で出来るが、介助なしには外出しない＞外出の頻度が少なく、日中も寝たり起きたりの生活をしている | 2 (0.2%) | 1 (0.9%) |
| ＜屋内での生活は何らかの介助を必要とし、日中もベッド上での生活が主であるが、座位を保つことができる＞車椅子に自分で乗り、食事、排泄はベッドから離れて行う | 3 (0.3%) | 2 (1.9%) |
| ＜屋内での生活は何らかの介助を必要とし、日中もベッド上での生活が主であるが、座位を保つことができる＞介助により車椅子に乗る | 1 (0.1%) | 0 (0%) |
| ＜1日中ベッド上で過ごし、排泄、食事、着替えの時に介助が要る＞自力で寝返りを打つ | 0 (0%) | 0 (0%) |
| ＜1日中ベッド上で過ごし、排泄、食事、着替えの時に介助が要る＞自力では寝返りも打たない | 5 (0.5%) | 0 (0%) |
| **昨年1年間の「身体の動かし方」についておたずねします。昨年1年間のうち、通常の時期の1日の時間の内訳を教えてください。通勤、仕事、家事などの時間をすべて含めてお答えください。余暇は含めません。: 1.座っている時間** | | |
| なかった | 32 (3.3%) | 0 (0%) |
| 1時間未満 | 23 (2.4%) | 7 (6.5%) |
| 1時間以上3時間未満 | 160 (16%) | 16 (15%) |
| 3時間以上5時間未満 | 283 (29%) | 30 (28%) |
| 5時間以上7時間未満 | 207 (21%) | 20 (19%) |
| 7時間以上9時間未満 | 146 (15%) | 18 (17%) |
| 9時間以上11時間未満 | 70 (7.2%) | 8 (7.4%) |
| 11時間以上 | 54 (5.5%) | 9 (8.3%) |
| **昨年1年間の「身体の動かし方」についておたずねします。昨年1年間のうち、通常の時期の1日の時間の内訳を教えてください。通勤、仕事、家事などの時間をすべて含めてお答えください。余暇は含めません。: 2.立っている時間** | | |
| なかった | 33 (3.4%) | 2 (1.9%) |
| 1時間未満 | 122 (13%) | 29 (27%) |
| 1時間以上3時間未満 | 334 (34%) | 47 (44%) |
| 3時間以上5時間未満 | 244 (25%) | 21 (19%) |
| 5時間以上7時間未満 | 136 (14%) | 2 (1.9%) |
| 7時間以上9時間未満 | 72 (7.4%) | 4 (3.7%) |
| 9時間以上11時間未満 | 21 (2.2%) | 0 (0%) |
| 11時間以上 | 13 (1.3%) | 3 (2.8%) |
| **昨年1年間の「身体の動かし方」についておたずねします。昨年1年間のうち、通常の時期の1日の時間の内訳を教えてください。通勤、仕事、家事などの時間をすべて含めてお答えください。余暇は含めません。: 3.歩いている時間** | | |

[1] Note: Items shown in this table were presented in Japanese in the original survey.

[2] Median (Q1, Q3); n (%)

| 質問項目[1] | 統合失調症なし N = 975[2] | 統合失調症あり N = 108[2] |
|---|---|---|
| なかった | 43 (4.4%) | 3 (2.8%) |
| 1時間未満 | 464 (48%) | 65 (60%) |
| 1時間以上3時間未満 | 348 (36%) | 34 (31%) |
| 3時間以上5時間未満 | 64 (6.6%) | 5 (4.6%) |
| 5時間以上7時間未満 | 33 (3.4%) | 0 (0%) |
| 7時間以上9時間未満 | 12 (1.2%) | 0 (0%) |
| 9時間以上11時間未満 | 4 (0.4%) | 0 (0%) |
| 11時間以上 | 7 (0.7%) | 1 (0.9%) |
| **昨年1年間の「身体の動かし方」についておたずねします。昨年1年間のうち、通常の時期の1日の時間の内訳を教えてください。通勤、仕事、家事などの時間をすべて含めてお答えください。余暇は含めません。: 4.力のいる作業をしている時間** | | |
| なかった | 393 (40%) | 55 (51%) |
| 1時間未満 | 429 (44%) | 42 (39%) |
| 1時間以上3時間未満 | 94 (9.6%) | 5 (4.6%) |
| 3時間以上5時間未満 | 30 (3.1%) | 4 (3.7%) |
| 5時間以上7時間未満 | 18 (1.8%) | 1 (0.9%) |
| 7時間以上9時間未満 | 10 (1.0%) | 0 (0%) |
| 9時間以上11時間未満 | 0 (0%) | 0 (0%) |
| 11時間以上 | 1 (0.1%) | 1 (0.9%) |
| **あなたの現在の状況について、以下の質問にお答えください。: 1.必要な時に、あなたの話を聞いてくれる人がいますか** | | |
| ほとんどいない | 70 (7.2%) | 15 (14%) |
| たまにいる | 171 (18%) | 26 (24%) |
| ときどきいる | 206 (21%) | 21 (19%) |
| よくいる | 273 (28%) | 28 (26%) |
| いつでもいる | 255 (26%) | 18 (17%) |
| **あなたの現在の状況について、以下の質問にお答えください。: 2.なにか困ったことがあった時、よいアドバイスをくれる人がいますか** | | |
| ほとんどいない | 96 (9.8%) | 16 (15%) |
| たまにいる | 188 (19%) | 28 (26%) |
| ときどきいる | 233 (24%) | 18 (17%) |
| よくいる | 243 (25%) | 32 (30%) |

[1] Note: Items shown in this table were presented in Japanese in the original survey.

[2] Median (Q1, Q3); n (%)

| **質問項目**[1] | **統合失調症なし**<br>N = 975[2] | **統合失調症あり**<br>N = 108[2] |
|---|---|---|
| いつでもいる | 215 (22%) | 14 (13%) |
| **あなたの現在の状況について、以下の質問にお答えください。: 3.あなたを心配したり、あなたに愛情をかけてくれる人はいますか** | | |
| ほとんどいない | 61 (6.3%) | 10 (9.3%) |
| たまにいる | 150 (15%) | 19 (18%) |
| ときどきいる | 192 (20%) | 25 (23%) |
| よくいる | 242 (25%) | 24 (22%) |
| いつでもいる | 330 (34%) | 30 (28%) |
| **あなたの現在の状況について、以下の質問にお答えください。: 4.日常の家事をしたり、手伝ってくれる人はいますか** | | |
| ほとんどいない | 213 (22%) | 23 (21%) |
| たまにいる | 150 (15%) | 22 (20%) |
| ときどきいる | 202 (21%) | 13 (12%) |
| よくいる | 192 (20%) | 22 (20%) |
| いつでもいる | 218 (22%) | 28 (26%) |
| **あなたの現在の状況について、以下の質問にお答えください。: 5.あなたに情緒的な支えを与えてくれるような人、(たとえば、あなたの直面する問題について相談できる人、難しい判断が必要な時に助けてくれる人)はいますか** | | |
| ほとんどいない | 105 (11%) | 19 (18%) |
| たまにいる | 154 (16%) | 21 (19%) |
| ときどきいる | 201 (21%) | 22 (20%) |
| よくいる | 240 (25%) | 23 (21%) |
| いつでもいる | 275 (28%) | 23 (21%) |
| **あなたの現在の状況について、以下の質問にお答えください。: 6.必要な時にいつでも連絡がとれる、親しくて、信頼・信用できる人はいますか** | | |
| ほとんどいない | 103 (11%) | 24 (22%) |
| たまにいる | 144 (15%) | 18 (17%) |
| ときどきいる | 180 (18%) | 16 (15%) |
| よくいる | 264 (27%) | 24 (22%) |
| いつでもいる | 284 (29%) | 26 (24%) |
| **あなたの現在の状況について、以下の質問にお答えください。: 1.気軽に個人的な相談ができる親しい友人は何人いますか** | | |
| 0人 | 225 (23%) | 36 (33%) |

[1] Note: Items shown in this table were presented in Japanese in the original survey.

[2] Median (Q1, Q3); n (%)

| 質問項目[1] | 統合失調症なし N = 975[2] | 統合失調症あり N = 108[2] |
|---|---|---|
| 1人 | 207 (21%) | 36 (33%) |
| 2人 | 258 (26%) | 21 (19%) |
| 3人以上 | 285 (29%) | 15 (14%) |
| **あなたの現在の状況について、以下の質問にお答えください。: 2.気軽に個人的な相談ができる親類は何人いますか** | | |
| 0人 | 195 (20%) | 37 (34%) |
| 1人 | 277 (28%) | 35 (32%) |
| 2人 | 258 (26%) | 16 (15%) |
| 3人以上 | 245 (25%) | 20 (19%) |
| **地域組織、自助集団、チャリティー、ボランティアグループや、宗教団体などの集まりにどれくらいの頻度で参加していますか。** | | |
| 全く／ほとんど参加しない | 858 (88%) | 90 (83%) |
| 時々参加する | 84 (8.6%) | 17 (16%) |
| 週に1回未満 | 12 (1.2%) | 1 (0.9%) |
| 週に1回以上 | 21 (2.2%) | 0 (0%) |
| **あなたは、生きがいがあると感じていますか。** | | |
| 非常にある | 148 (15%) | 10 (9.3%) |
| ある | 480 (49%) | 38 (35%) |
| あまりない | 273 (28%) | 43 (40%) |
| 全くない | 74 (7.6%) | 17 (16%) |
| **あなたはご自分がどれくらい幸せだと感じていますか。** | | |
| 大変幸せ | 158 (16%) | 12 (11%) |
| 幸せ | 539 (55%) | 44 (41%) |
| どちらとも言えない | 213 (22%) | 29 (27%) |
| 幸せでない | 65 (6.7%) | 23 (21%) |
| **最近1週間の体や心の状態について、お聞きします。それぞれ最もあてはまる選択肢1つ選択してください。: 1.食べたくない。食欲がおちた** | | |
| 全く／ほとんどなかった | 772 (79%) | 63 (58%) |
| たまにあった（1〜2日） | 154 (16%) | 28 (26%) |
| しばしばあった（3〜4日） | 23 (2.4%) | 7 (6.5%) |
| いつもあった（ほぼ毎日） | 26 (2.7%) | 10 (9.3%) |

[1] Note: Items shown in this table were presented in Japanese in the original survey.

[2] Median (Q1, Q3); n (%)

| 質問項目[1] | 統合失調症なし<br>N = 975[2] | 統合失調症あり<br>N = 108[2] |
|---|---|---|
| **最近1週間の体や心の状態について、お聞きします。それぞれ最もあてはまる選択肢1つ選択してください。: 2.ゆううつだ** | | |
| 全く／ほとんどなかった | 536 (55%) | 22 (20%) |
| たまにあった（1〜2日） | 300 (31%) | 41 (38%) |
| しばしばあった（3〜4日） | 83 (8.5%) | 26 (24%) |
| いつもあった（ほぼ毎日） | 56 (5.7%) | 19 (18%) |
| **最近1週間の体や心の状態について、お聞きします。それぞれ最もあてはまる選択肢1つ選択してください。: 3.何をするのも面倒だ** | | |
| 全く／ほとんどなかった | 440 (45%) | 14 (13%) |
| たまにあった（1〜2日） | 359 (37%) | 41 (38%) |
| しばしばあった（3〜4日） | 108 (11%) | 27 (25%) |
| いつもあった（ほぼ毎日） | 68 (7.0%) | 26 (24%) |
| **最近1週間の体や心の状態について、お聞きします。それぞれ最もあてはまる選択肢1つ選択してください。: 4.なかなか眠れない** | | |
| 全く／ほとんどなかった | 562 (58%) | 30 (28%) |
| たまにあった（1〜2日） | 294 (30%) | 37 (34%) |
| しばしばあった（3〜4日） | 83 (8.5%) | 22 (20%) |
| いつもあった（ほぼ毎日） | 36 (3.7%) | 19 (18%) |
| **最近1週間の体や心の状態について、お聞きします。それぞれ最もあてはまる選択肢1つ選択してください。: 5.生活について満足して過ごせる** | | |
| 全く／ほとんどなかった | 232 (24%) | 24 (22%) |
| たまにあった（1〜2日） | 230 (24%) | 33 (31%) |
| しばしばあった（3〜4日） | 245 (25%) | 36 (33%) |
| いつもあった（ほぼ毎日） | 268 (27%) | 15 (14%) |
| **最近1週間の体や心の状態について、お聞きします。それぞれ最もあてはまる選択肢1つ選択してください。: 6.一人ぼっちでさびしい** | | |
| 全く／ほとんどなかった | 710 (73%) | 55 (51%) |
| たまにあった（1〜2日） | 182 (19%) | 33 (31%) |
| しばしばあった（3〜4日） | 59 (6.1%) | 12 (11%) |
| いつもあった（ほぼ毎日） | 24 (2.5%) | 8 (7.4%) |
| **最近1週間の体や心の状態について、お聞きします。それぞれ最もあてはまる選択肢1つ選択してください。: 7.皆がよそよそしいと思う** | | |
| 全く／ほとんどなかった | 786 (81%) | 59 (55%) |

[1] Note: Items shown in this table were presented in Japanese in the original survey.

[2] Median (Q1, Q3); n (%)

| 質問項目[1] | 統合失調症なし N = 975[2] | 統合失調症あり N = 108[2] |
|---|---|---|
| たまにあった（1〜2日） | 130 (13%) | 33 (31%) |
| しばしばあった（3〜4日） | 41 (4.2%) | 8 (7.4%) |
| いつもあった（ほぼ毎日） | 18 (1.8%) | 8 (7.4%) |
| **最近1週間の体や心の状態について、お聞きします。それぞれ最もあてはまる選択肢1つ選択してください。: 8.毎日が楽しい** | | |
| 全く／ほとんどなかった | 194 (20%) | 30 (28%) |
| たまにあった（1〜2日） | 300 (31%) | 39 (36%) |
| しばしばあった（3〜4日） | 288 (30%) | 28 (26%) |
| いつもあった（ほぼ毎日） | 193 (20%) | 11 (10%) |
| **最近1週間の体や心の状態について、お聞きします。それぞれ最もあてはまる選択肢1つ選択してください。: 9.悲しいと感じる** | | |
| 全く／ほとんどなかった | 568 (58%) | 23 (21%) |
| たまにあった（1〜2日） | 295 (30%) | 45 (42%) |
| しばしばあった（3〜4日） | 81 (8.3%) | 24 (22%) |
| いつもあった（ほぼ毎日） | 31 (3.2%) | 16 (15%) |
| **最近1週間の体や心の状態について、お聞きします。それぞれ最もあてはまる選択肢1つ選択してください。: 10.皆が自分を嫌っていると感じる** | | |
| 全く／ほとんどなかった | 762 (78%) | 41 (38%) |
| たまにあった（1〜2日） | 141 (14%) | 39 (36%) |
| しばしばあった（3〜4日） | 46 (4.7%) | 14 (13%) |
| いつもあった（ほぼ毎日） | 26 (2.7%) | 14 (13%) |
| **最近1週間の体や心の状態について、お聞きします。それぞれ最もあてはまる選択肢1つ選択してください。: 11.仕事が手につかない** | | |
| 全く／ほとんどなかった | 756 (78%) | 47 (44%) |
| たまにあった（1〜2日） | 155 (16%) | 33 (31%) |
| しばしばあった（3〜4日） | 50 (5.1%) | 14 (13%) |
| いつもあった（ほぼ毎日） | 14 (1.4%) | 14 (13%) |
| **あなたの気持ち、考えなどについてお伺いします。: 1.過去1か月、「人生での大切な事が自分の思うようにならない」と感じましたか** | | |
| 全くない | 278 (29%) | 4 (3.7%) |
| ほとんどない | 298 (31%) | 14 (13%) |
| ときどき | 303 (31%) | 52 (48%) |

[1] Note: Items shown in this table were presented in Japanese in the original survey.

[2] Median (Q1, Q3); n (%)

| 質問項目[1] | 統合失調症なし<br>N = 975[2] | 統合失調症あり<br>N = 108[2] |
|---|---|---|
| 頻繁に | 58 (5.9%) | 18 (17%) |
| とても頻繁に | 38 (3.9%) | 20 (19%) |
| **あなたの気持ち、考えなどについてお伺いします。: 2.過去1か月、「自分の問題を解決する能力に自信がある」と感じましたか** | | |
| 全くない | 186 (19%) | 35 (32%) |
| ほとんどない | 272 (28%) | 35 (32%) |
| ときどき | 375 (38%) | 27 (25%) |
| 頻繁に | 111 (11%) | 10 (9.3%) |
| とても頻繁に | 31 (3.2%) | 1 (0.9%) |
| **あなたの気持ち、考えなどについてお伺いします。: 3.過去1か月、「思うように物事がいっている」と感じましたか** | | |
| 全くない | 148 (15%) | 26 (24%) |
| ほとんどない | 220 (23%) | 32 (30%) |
| ときどき | 463 (47%) | 43 (40%) |
| 頻繁に | 124 (13%) | 5 (4.6%) |
| とても頻繁に | 20 (2.1%) | 2 (1.9%) |
| **あなたの気持ち、考えなどについてお伺いします。: 4.過去1か月、「多くの困難が山積みで自分の手に負えない」と感じましたか** | | |
| 全くない | 280 (29%) | 9 (8.3%) |
| ほとんどない | 360 (37%) | 31 (29%) |
| ときどき | 254 (26%) | 33 (31%) |
| 頻繁に | 51 (5.2%) | 19 (18%) |
| とても頻繁に | 30 (3.1%) | 16 (15%) |
| **あなたの周りの状況について、以下の質問にお答えください。: 1.一般的に、人は信用できると思いますか** | | |
| 全く思わない | 83 (8.5%) | 18 (17%) |
| あまり思わない | 355 (36%) | 49 (45%) |
| 思う | 503 (52%) | 37 (34%) |
| 非常によく思う | 34 (3.5%) | 4 (3.7%) |
| **あなたの周りの状況について、以下の質問にお答えください。: 2.多くの人は隙さえあれば、他の人を利用しようとするものだと思いますか** | | |
| 全く思わない | 78 (8.0%) | 1 (0.9%) |

[1] Note: Items shown in this table were presented in Japanese in the original survey.

[2] Median (Q1, Q3); n (%)

| 質問項目[1] | 統合失調症なし<br>N = 975[2] | 統合失調症あり<br>N = 108[2] |
|---|---|---|
| あまり思わない | 542 (56%) | 49 (45%) |
| 思う | 318 (33%) | 49 (45%) |
| 非常によく思う | 37 (3.8%) | 9 (8.3%) |
| **あなたの周りの状況について、以下の質問にお答えください。: 3.多くの場合、人は他の人の役に立とうとすると思いますか** | | |
| 全く思わない | 51 (5.2%) | 6 (5.6%) |
| あまり思わない | 338 (35%) | 44 (41%) |
| 思う | 560 (57%) | 55 (51%) |
| 非常によく思う | 26 (2.7%) | 3 (2.8%) |
| **学校教育はどのくらいまで受けられましたか。1つだけ選択してください。** | | |
| 中学校 | 11 (1.1%) | 6 (5.6%) |
| 高校 | 234 (24%) | 35 (32%) |
| 短大卒・専門学校・4年制大学中退 | 297 (30%) | 34 (31%) |
| 大学以上 | 427 (44%) | 31 (29%) |
| その他 | 6 (0.6%) | 2 (1.9%) |
| **現在の従事している職業は何ですか。** | | |
| 無職 | 66 (6.8%) | 36 (33%) |
| 主婦（主夫） | 257 (26%) | 29 (27%) |
| 専門的・技術的職業従事者 | 94 (9.6%) | 4 (3.7%) |
| 管理的職業従事者 | 6 (0.6%) | 0 (0%) |
| 事務従事者 | 226 (23%) | 10 (9.3%) |
| 販売従事者 | 48 (4.9%) | 7 (6.5%) |
| サービス職業従事者 | 108 (11%) | 4 (3.7%) |
| 保安職業従事者 | 0 (0%) | 0 (0%) |
| 農業漁業作業者 | 2 (0.2%) | 0 (0%) |
| 運輸・通信従事者 | 7 (0.7%) | 0 (0%) |
| 生産工程・労務作業者 | 26 (2.7%) | 6 (5.6%) |
| その他の職業 | 135 (14%) | 12 (11%) |
| **現在従事されているお仕事の雇用形態は何ですか。** | | |
| 正社員・職員 | 343 (53%) | 5 (12%) |
| 契約社員・職員 | 49 (7.5%) | 2 (4.7%) |

[1] Note: Items shown in this table were presented in Japanese in the original survey.

[2] Median (Q1, Q3); n (%)

| 質問項目[1] | 統合失調症なし<br>N = 975[2] | 統合失調症あり<br>N = 108[2] |
|---|---|---|
| 派遣社員・職員 | 29 (4.4%) | 3 (7.0%) |
| パート・アルバイト | 196 (30%) | 29 (67%) |
| 自営・経営者 | 35 (5.4%) | 4 (9.3%) |
| 回答なし | 323 | 65 |
| **現在の世帯年収（税込み）はどのくらいですか。** | | |
| 0〜299万円 | 211 (22%) | 47 (44%) |
| 300〜599万円 | 375 (38%) | 40 (37%) |
| 600〜899万円 | 231 (24%) | 16 (15%) |
| 900〜1,199万円 | 97 (9.9%) | 4 (3.7%) |
| 1,200〜1,499万円 | 38 (3.9%) | 1 (0.9%) |
| 1,500万円以上 | 23 (2.4%) | 0 (0%) |

[1] Note: Items shown in this table were presented in Japanese in the original survey.

[2] Median (Q1, Q3); n (%)

**Table S3 Descriptive statistics of demographic characteristics and key study variables stratified by age in participants with schizophrenia**

| **質問項目**[1] | **35歳以上**<br>N = 195[2] | **35歳未満**<br>N = 28[2] |
|---|---|---|
| **年齢** | 48 (43, 54) | 33 (29, 33) |
| **性別** | | |
| 男性 | 107 (55%) | 8 (29%) |
| 女性 | 88 (45%) | 20 (71%) |
| **あなたの現在の身長および体重をお答えください。（半角数字でご記入ください）※小数点以下は四捨五入してください。身長（cm）** | 165 (160, 172) | 160 (156, 167) |
| **あなたの現在の身長および体重をお答えください。（半角数字でご記入ください）※小数点以下は四捨五入してください。体重（kg）** | 68 (59, 80) | 58 (49, 73) |
| **現在の結婚状況についてお聞きします。1つだけ選択してください。** | | |
| 結婚・再婚・内縁（いずれも別居は除く） | 55 (28%) | 7 (25%) |
| 離婚 | 20 (10%) | 0 (0%) |
| 別居 | 3 (1.5%) | 0 (0%) |
| 死別 | 2 (1.0%) | 0 (0%) |
| 未婚 | 115 (59%) | 21 (75%) |
| その他 | 0 (0%) | 0 (0%) |
| **現在、どなたと一緒にお住まいですか。同居しているすべての人を選択してください。（いくつでも）（同居・配偶者）** | | |
| なし | 140 (72%) | 21 (75%) |
| あり | 55 (28%) | 7 (25%) |
| **現在、どなたと一緒にお住まいですか。同居しているすべての人を選択してください。（いくつでも）（同居・子供）** | | |
| なし | 152 (78%) | 24 (86%) |
| あり | 43 (22%) | 4 (14%) |
| **現在、どなたと一緒にお住まいですか。同居しているすべての人を選択してください。（いくつでも）（同居・親）** | | |
| なし | 92 (47%) | 8 (29%) |
| あり | 103 (53%) | 20 (71%) |
| **現在、どなたと一緒にお住まいですか。同居しているすべての人を選択してください。（いくつでも）（同居・その他）** | | |
| なし | 157 (81%) | 22 (79%) |

[1] Note: Items shown in this table were presented in Japanese in the original survey.

[2] Median (Q1, Q3); n (%)

| **質問項目**[1] | **35歳以上**<br>N = 195[2] | **35歳未満**<br>N = 28[2] |
|---|---|---|
| あり | 38 (19%) | 6 (21%) |
| **現在、どなたと一緒にお住まいですか。同居しているすべての人を選択してください。（いくつでも）（同居・独り暮らし）** | | |
| なし | 162 (83%) | 28 (100%) |
| あり | 33 (17%) | 0 (0%) |
| **現在の同居人数は何人ですか。（半角数字でご記入ください）※あなたは含めません。** | | |
| 1 | 47 (29%) | 5 (18%) |
| 2 | 63 (39%) | 10 (36%) |
| 3 | 32 (20%) | 11 (39%) |
| 4 | 14 (8.6%) | 1 (3.6%) |
| 5 | 4 (2.5%) | 1 (3.6%) |
| 6 | 1 (0.6%) | 0 (0%) |
| 8 | 1 (0.6%) | 0 (0%) |
| 回答なし | 33 | 0 |
| **現在の同居人数は何人ですか。（半角数字でご記入ください）※あなたは含めません。（年齢14歳未満の同居人数）** | | |
| 0 | 20 (47%) | 0 (0%) |
| 1 | 14 (33%) | 3 (75%) |
| 2 | 9 (21%) | 1 (25%) |
| 回答なし | 152 | 24 |
| **全体的にみて、あなたの過去1か月間の健康状態はいかがでしたか。** | | |
| 最高に良い | 5 (2.6%) | 1 (3.6%) |
| やや良い | 30 (15%) | 3 (11%) |
| 良い | 66 (34%) | 10 (36%) |
| あまり良くない | 74 (38%) | 10 (36%) |
| 良くない | 20 (10%) | 4 (14%) |
| **現在の日常生活の状態はいかがですか。最も当てはまると思われるものを選んでください。** | | |
| 身体に特に障害はない | 116 (59%) | 18 (64%) |
| ＜身体に何らかの障害はあるが、日常生活はほぼ自分で出来、独力で外出する＞交通機関などを利用して外出する | 33 (17%) | 3 (11%) |
| ＜身体に何らかの障害はあるが、日常生活はほぼ自分で出来、独力で外出する＞隣近所にのみ出かける | 33 (17%) | 6 (21%) |
| ＜屋内での生活はおおむね自分で出来るが、介助なしには外出しない＞介助をしてもらって外出し、日中はほとんどベッドから離れて生活する | 5 (2.6%) | 0 (0%) |

[1] Note: Items shown in this table were presented in Japanese in the original survey.

[2] Median (Q1, Q3); n (%)

| 質問項目[1] | 35歳以上<br>N = 195[2] | 35歳未満<br>N = 28[2] |
|---|---|---|
| ＜屋内での生活はおおむね自分で出来るが、介助なしには外出しない＞外出の頻度が少なく、日中も寝たり起きたりの生活をしている | 4 (2.1%) | 1 (3.6%) |
| ＜屋内での生活は何らかの介助を必要とし、日中もベッド上での生活が主であるが、座位を保つことができる＞車椅子に自分で乗り、食事、排泄はベッドから離れて行う | 3 (1.5%) | 0 (0%) |
| ＜屋内での生活は何らかの介助を必要とし、日中もベッド上での生活が主であるが、座位を保つことができる＞介助により車椅子に乗る | 1 (0.5%) | 0 (0%) |
| ＜1日中ベッド上で過ごし、排泄、食事、着替えの時に介助が要る＞自力で寝返りを打つ | 0 (0%) | 0 (0%) |
| ＜1日中ベッド上で過ごし、排泄、食事、着替えの時に介助が要る＞自力では寝返りも打たない | 0 (0%) | 0 (0%) |
| **昨年1年間の「身体の動かし方」についておたずねします。昨年1年間のうち、通常の時期の1日の時間の内訳を教えてください。通勤、仕事、家事などの時間をすべて含めてお答えください。余暇は含めません。：1.座っている時間** | | |
| なかった | 0 (0%) | 0 (0%) |
| 1時間未満 | 15 (7.7%) | 1 (3.6%) |
| 1時間以上3時間未満 | 34 (17%) | 7 (25%) |
| 3時間以上5時間未満 | 49 (25%) | 6 (21%) |
| 5時間以上7時間未満 | 40 (21%) | 5 (18%) |
| 7時間以上9時間未満 | 21 (11%) | 7 (25%) |
| 9時間以上11時間未満 | 14 (7.2%) | 1 (3.6%) |
| 11時間以上 | 22 (11%) | 1 (3.6%) |
| **昨年1年間の「身体の動かし方」についておたずねします。昨年1年間のうち、通常の時期の1日の時間の内訳を教えてください。通勤、仕事、家事などの時間をすべて含めてお答えください。余暇は含めません。：2.立っている時間** | | |
| なかった | 7 (3.6%) | 1 (3.6%) |
| 1時間未満 | 60 (31%) | 6 (21%) |
| 1時間以上3時間未満 | 76 (39%) | 14 (50%) |
| 3時間以上5時間未満 | 29 (15%) | 5 (18%) |
| 5時間以上7時間未満 | 10 (5.1%) | 0 (0%) |
| 7時間以上9時間未満 | 7 (3.6%) | 1 (3.6%) |
| 9時間以上11時間未満 | 3 (1.5%) | 0 (0%) |
| 11時間以上 | 3 (1.5%) | 1 (3.6%) |
| **昨年1年間の「身体の動かし方」についておたずねします。昨年1年間のうち、通常の時期の1日の時間の内訳を教えてください。通勤、仕事、家事などの時間をすべて含めてお答えください。余暇は含めません。：3.歩いている時間** | | |
| なかった | 8 (4.1%) | 2 (7.1%) |
| 1時間未満 | 111 (57%) | 13 (46%) |

[1] Note: Items shown in this table were presented in Japanese in the original survey.

[2] Median (Q1, Q3); n (%)

| 質問項目[1] | 35歳以上<br>N = 195[2] | 35歳未満<br>N = 28[2] |
|---|---|---|
| 1時間以上3時間未満 | 69 (35%) | 9 (32%) |
| 3時間以上5時間未満 | 5 (2.6%) | 3 (11%) |
| 5時間以上7時間未満 | 0 (0%) | 0 (0%) |
| 7時間以上9時間未満 | 1 (0.5%) | 0 (0%) |
| 9時間以上11時間未満 | 1 (0.5%) | 0 (0%) |
| 11時間以上 | 0 (0%) | 1 (3.6%) |
| **昨年1年間の「身体の動かし方」についておたずねします。昨年1年間のうち、通常の時期の1日の時間の内訳を教えてください。通勤、仕事、家事などの時間をすべて含めてお答えください。余暇は含めません。: 4.力のいる作業をしている時間** | | |
| なかった | 101 (52%) | 15 (54%) |
| 1時間未満 | 70 (36%) | 8 (29%) |
| 1時間以上3時間未満 | 13 (6.7%) | 2 (7.1%) |
| 3時間以上5時間未満 | 6 (3.1%) | 2 (7.1%) |
| 5時間以上7時間未満 | 1 (0.5%) | 0 (0%) |
| 7時間以上9時間未満 | 2 (1.0%) | 0 (0%) |
| 9時間以上11時間未満 | 1 (0.5%) | 0 (0%) |
| 11時間以上 | 1 (0.5%) | 1 (3.6%) |
| **あなたの現在の状況について、以下の質問にお答えください。: 1.必要な時に、あなたの話を聞いてくれる人がいますか** | | |
| ほとんどいない | 31 (16%) | 3 (11%) |
| たまにいる | 44 (23%) | 5 (18%) |
| ときどきいる | 44 (23%) | 4 (14%) |
| よくいる | 39 (20%) | 8 (29%) |
| いつでもいる | 37 (19%) | 8 (29%) |
| **あなたの現在の状況について、以下の質問にお答えください。: 2.なにか困ったことがあった時、よいアドバイスをくれる人がいますか** | | |
| ほとんどいない | 36 (18%) | 4 (14%) |
| たまにいる | 49 (25%) | 4 (14%) |
| ときどきいる | 37 (19%) | 4 (14%) |
| よくいる | 45 (23%) | 9 (32%) |
| いつでもいる | 28 (14%) | 7 (25%) |
| **あなたの現在の状況について、以下の質問にお答えください。: 3.あなたを心配したり、あなたに愛情をかけてくれる人はいますか** | | |

[1] Note: Items shown in this table were presented in Japanese in the original survey.

[2] Median (Q1, Q3); n (%)

| 質問項目[1] | 35歳以上<br>N = 195[2] | 35歳未満<br>N = 28[2] |
|---|---|---|
| ほとんどいない | 25 (13%) | 2 (7.1%) |
| たまにいる | 33 (17%) | 6 (21%) |
| ときどきいる | 53 (27%) | 3 (11%) |
| よくいる | 38 (19%) | 5 (18%) |
| いつでもいる | 46 (24%) | 12 (43%) |
| **あなたの現在の状況について、以下の質問にお答えください。: 4.日常の家事をしたり、手伝ってくれる人はいますか** | | |
| ほとんどいない | 39 (20%) | 4 (14%) |
| たまにいる | 36 (18%) | 5 (18%) |
| ときどきいる | 23 (12%) | 2 (7.1%) |
| よくいる | 43 (22%) | 7 (25%) |
| いつでもいる | 54 (28%) | 10 (36%) |
| **あなたの現在の状況について、以下の質問にお答えください。: 5.あなたに情緒的な支えを与えてくれるような人、（たとえば、あなたの直面する問題について相談できる人、難しい判断が必要な時に助けてくれる人）はいますか** | | |
| ほとんどいない | 39 (20%) | 4 (14%) |
| たまにいる | 40 (21%) | 4 (14%) |
| ときどきいる | 41 (21%) | 4 (14%) |
| よくいる | 35 (18%) | 8 (29%) |
| いつでもいる | 40 (21%) | 8 (29%) |
| **あなたの現在の状況について、以下の質問にお答えください。: 6.必要な時にいつでも連絡がとれる、親しくて、信頼・信用できる人はいますか** | | |
| ほとんどいない | 41 (21%) | 6 (21%) |
| たまにいる | 37 (19%) | 3 (11%) |
| ときどきいる | 39 (20%) | 2 (7.1%) |
| よくいる | 36 (18%) | 7 (25%) |
| いつでもいる | 42 (22%) | 10 (36%) |
| **あなたの現在の状況について、以下の質問にお答えください。: 1.気軽に個人的な相談ができる親しい友人は何人いますか** | | |
| 0人 | 80 (41%) | 12 (43%) |
| 1人 | 52 (27%) | 9 (32%) |
| 2人 | 36 (18%) | 2 (7.1%) |
| 3人以上 | 27 (14%) | 5 (18%) |

[1] Note: Items shown in this table were presented in Japanese in the original survey.

[2] Median (Q1, Q3); n (%)

| 質問項目[1] | 35歳以上<br>N = 195[2] | 35歳未満<br>N = 28[2] |
|---|---|---|
| **あなたの現在の状況について、以下の質問にお答えください。: 2.気軽に個人的な相談ができる親類は何人いますか** | | |
| 0人 | 65 (33%) | 8 (29%) |
| 1人 | 62 (32%) | 5 (18%) |
| 2人 | 29 (15%) | 11 (39%) |
| 3人以上 | 39 (20%) | 4 (14%) |
| **地域組織、自助集団、チャリティー、ボランティアグループや、宗教団体などの集まりにどれくらいの頻度で参加していますか。** | | |
| 全く／ほとんど参加しない | 164 (84%) | 25 (89%) |
| 時々参加する | 19 (9.7%) | 3 (11%) |
| 週に1回未満 | 5 (2.6%) | 0 (0%) |
| 週に1回以上 | 7 (3.6%) | 0 (0%) |
| **あなたは、生きがいがあると感じていますか。** | | |
| 非常にある | 17 (8.7%) | 7 (25%) |
| ある | 65 (33%) | 8 (29%) |
| あまりない | 81 (42%) | 8 (29%) |
| 全くない | 32 (16%) | 5 (18%) |
| **あなたはご自分がどれくらい幸せだと感じていますか。** | | |
| 大変幸せ | 16 (8.2%) | 6 (21%) |
| 幸せ | 66 (34%) | 10 (36%) |
| どちらとも言えない | 68 (35%) | 4 (14%) |
| 幸せでない | 45 (23%) | 8 (29%) |
| **最近1週間の体や心の状態について、お聞きします。それぞれ最もあてはまる選択肢1つ選択してください。: 1.食べたくない。食欲がおちた** | | |
| 全く／ほとんどなかった | 130 (67%) | 20 (71%) |
| たまにあった（1～2日） | 42 (22%) | 5 (18%) |
| しばしばあった（3～4日） | 12 (6.2%) | 0 (0%) |
| いつもあった（ほぼ毎日） | 11 (5.6%) | 3 (11%) |
| **最近1週間の体や心の状態について、お聞きします。それぞれ最もあてはまる選択肢1つ選択してください。: 2.ゆううつだ** | | |
| 全く／ほとんどなかった | 46 (24%) | 5 (18%) |
| たまにあった（1～2日） | 72 (37%) | 12 (43%) |

[1] Note: Items shown in this table were presented in Japanese in the original survey.

[2] Median (Q1, Q3); n (%)

| 質問項目[1] | 35歳以上 N = 195[2] | 35歳未満 N = 28[2] |
|---|---|---|
| しばしばあった（3〜4日） | 42 (22%) | 3 (11%) |
| いつもあった（ほぼ毎日） | 35 (18%) | 8 (29%) |
| **最近1週間の体や心の状態について、お聞きします。それぞれ最もあてはまる選択肢1つ選択してください。: 3.何をするのも面倒だ** | | |
| 全く／ほとんどなかった | 34 (17%) | 5 (18%) |
| たまにあった（1〜2日） | 77 (39%) | 9 (32%) |
| しばしばあった（3〜4日） | 37 (19%) | 8 (29%) |
| いつもあった（ほぼ毎日） | 47 (24%) | 6 (21%) |
| **最近1週間の体や心の状態について、お聞きします。それぞれ最もあてはまる選択肢1つ選択してください。: 4.なかなか眠れない** | | |
| 全く／ほとんどなかった | 69 (35%) | 7 (25%) |
| たまにあった（1〜2日） | 53 (27%) | 12 (43%) |
| しばしばあった（3〜4日） | 33 (17%) | 4 (14%) |
| いつもあった（ほぼ毎日） | 40 (21%) | 5 (18%) |
| **最近1週間の体や心の状態について、お聞きします。それぞれ最もあてはまる選択肢1つ選択してください。: 5.生活について満足して過ごせる** | | |
| 全く／ほとんどなかった | 52 (27%) | 6 (21%) |
| たまにあった（1〜2日） | 63 (32%) | 5 (18%) |
| しばしばあった（3〜4日） | 49 (25%) | 10 (36%) |
| いつもあった（ほぼ毎日） | 31 (16%) | 7 (25%) |
| **最近1週間の体や心の状態について、お聞きします。それぞれ最もあてはまる選択肢1つ選択してください。: 6.一人ぼっちでさびしい** | | |
| 全く／ほとんどなかった | 102 (52%) | 17 (61%) |
| たまにあった（1〜2日） | 56 (29%) | 8 (29%) |
| しばしばあった（3〜4日） | 22 (11%) | 2 (7.1%) |
| いつもあった（ほぼ毎日） | 15 (7.7%) | 1 (3.6%) |
| **最近1週間の体や心の状態について、お聞きします。それぞれ最もあてはまる選択肢1つ選択してください。: 7.皆がよそよそしいと思う** | | |
| 全く／ほとんどなかった | 108 (55%) | 17 (61%) |
| たまにあった（1〜2日） | 51 (26%) | 9 (32%) |
| しばしばあった（3〜4日） | 21 (11%) | 1 (3.6%) |
| いつもあった（ほぼ毎日） | 15 (7.7%) | 1 (3.6%) |

[1] Note: Items shown in this table were presented in Japanese in the original survey.

[2] Median (Q1, Q3); n (%)

| 質問項目[1] | 35歳以上<br>N = 195[2] | 35歳未満<br>N = 28[2] |
|---|---|---|
| **最近1週間の体や心の状態について、お聞きします。それぞれ最もあてはまる選択肢1つ選択してください。: 8.毎日が楽しい** | | |
| 全く／ほとんどなかった | 63 (32%) | 8 (29%) |
| たまにあった（1〜2日） | 73 (37%) | 11 (39%) |
| しばしばあった（3〜4日） | 42 (22%) | 4 (14%) |
| いつもあった（ほぼ毎日） | 17 (8.7%) | 5 (18%) |
| **最近1週間の体や心の状態について、お聞きします。それぞれ最もあてはまる選択肢1つ選択してください。: 9.悲しいと感じる** | | |
| 全く／ほとんどなかった | 60 (31%) | 6 (21%) |
| たまにあった（1〜2日） | 79 (41%) | 7 (25%) |
| しばしばあった（3〜4日） | 36 (18%) | 8 (29%) |
| いつもあった（ほぼ毎日） | 20 (10%) | 7 (25%) |
| **最近1週間の体や心の状態について、お聞きします。それぞれ最もあてはまる選択肢1つ選択してください。: 10.皆が自分を嫌っていると感じる** | | |
| 全く／ほとんどなかった | 80 (41%) | 16 (57%) |
| たまにあった（1〜2日） | 62 (32%) | 6 (21%) |
| しばしばあった（3〜4日） | 24 (12%) | 4 (14%) |
| いつもあった（ほぼ毎日） | 29 (15%) | 2 (7.1%) |
| **最近1週間の体や心の状態について、お聞きします。それぞれ最もあてはまる選択肢1つ選択してください。: 11.仕事が手につかない** | | |
| 全く／ほとんどなかった | 86 (44%) | 15 (54%) |
| たまにあった（1〜2日） | 57 (29%) | 7 (25%) |
| しばしばあった（3〜4日） | 20 (10%) | 4 (14%) |
| いつもあった（ほぼ毎日） | 32 (16%) | 2 (7.1%) |
| **あなたの気持ち、考えなどについてお伺いします。: 1.過去1か月、「人生での大切な事が自分の思うようにならない」と感じましたか** | | |
| 全くない | 10 (5.1%) | 4 (14%) |
| ほとんどない | 34 (17%) | 3 (11%) |
| ときどき | 80 (41%) | 9 (32%) |
| 頻繁に | 34 (17%) | 6 (21%) |
| とても頻繁に | 37 (19%) | 6 (21%) |
| **あなたの気持ち、考えなどについてお伺いします。: 2.過去1か月、「自分の問題を解決する能力に自信がある」と感じましたか** | | |

[1] Note: Items shown in this table were presented in Japanese in the original survey.

[2] Median (Q1, Q3); n (%)

| 質問項目[1] | 35歳以上 N = 195[2] | 35歳未満 N = 28[2] |
|---|---|---|
| 全くない | 54 (28%) | 12 (43%) |
| ほとんどない | 65 (33%) | 7 (25%) |
| ときどき | 59 (30%) | 6 (21%) |
| 頻繁に | 15 (7.7%) | 3 (11%) |
| とても頻繁に | 2 (1.0%) | 0 (0%) |
| **あなたの気持ち、考えなどについてお伺いします。: 3.過去1か月、「思うように物事がいっている」と感じましたか** | | |
| 全くない | 44 (23%) | 8 (29%) |
| ほとんどない | 67 (34%) | 8 (29%) |
| ときどき | 69 (35%) | 9 (32%) |
| 頻繁に | 12 (6.2%) | 2 (7.1%) |
| とても頻繁に | 3 (1.5%) | 1 (3.6%) |
| **あなたの気持ち、考えなどについてお伺いします。: 4.過去1か月、「多くの困難が山積みで自分の手に負えない」と感じましたか** | | |
| 全くない | 20 (10%) | 2 (7.1%) |
| ほとんどない | 60 (31%) | 6 (21%) |
| ときどき | 59 (30%) | 10 (36%) |
| 頻繁に | 29 (15%) | 6 (21%) |
| とても頻繁に | 27 (14%) | 4 (14%) |
| **あなたの周りの状況について、以下の質問にお答えください。: 1.一般的に、人は信用できると思いますか** | | |
| 全く思わない | 31 (16%) | 6 (21%) |
| あまり思わない | 94 (48%) | 10 (36%) |
| 思う | 67 (34%) | 8 (29%) |
| 非常によく思う | 3 (1.5%) | 4 (14%) |
| **あなたの周りの状況について、以下の質問にお答えください。: 2.多くの人は隙さえあれば、他の人を利用しようとするものだと思いますか** | | |
| 全く思わない | 8 (4.1%) | 1 (3.6%) |
| あまり思わない | 87 (45%) | 10 (36%) |
| 思う | 82 (42%) | 13 (46%) |
| 非常によく思う | 18 (9.2%) | 4 (14%) |
| **あなたの周りの状況について、以下の質問にお答えください。: 3.多くの場合、人は他の人の役に立とうとすると思いますか** | | |
| 全く思わない | 14 (7.2%) | 3 (11%) |

[1] Note: Items shown in this table were presented in Japanese in the original survey.

[2] Median (Q1, Q3); n (%)

| 質問項目[1] | 35歳以上<br>N = 195[2] | 35歳未満<br>N = 28[2] |
|---|---|---|
| あまり思わない | 85 (44%) | 9 (32%) |
| 思う | 89 (46%) | 14 (50%) |
| 非常によく思う | 7 (3.6%) | 2 (7.1%) |
| **学校教育はどのくらいまで受けられましたか。1つだけ選択してください。** | | |
| 中学校 | 10 (5.1%) | 3 (11%) |
| 高校 | 67 (34%) | 11 (39%) |
| 短大卒・専門学校・4年制大学中退 | 50 (26%) | 1 (3.6%) |
| 大学以上 | 67 (34%) | 11 (39%) |
| その他 | 1 (0.5%) | 2 (7.1%) |
| **現在の従事している職業は何ですか。** | | |
| 無職 | 85 (44%) | 9 (32%) |
| 主婦（主夫） | 28 (14%) | 4 (14%) |
| 専門的・技術的職業従事者 | 7 (3.6%) | 2 (7.1%) |
| 管理的職業従事者 | 3 (1.5%) | 0 (0%) |
| 事務従事者 | 13 (6.7%) | 4 (14%) |
| 販売従事者 | 8 (4.1%) | 0 (0%) |
| サービス職業従事者 | 7 (3.6%) | 2 (7.1%) |
| 保安職業従事者 | 0 (0%) | 0 (0%) |
| 農業漁業作業者 | 2 (1.0%) | 0 (0%) |
| 運輸・通信従事者 | 2 (1.0%) | 0 (0%) |
| 生産工程・労務作業者 | 14 (7.2%) | 2 (7.1%) |
| その他の職業 | 26 (13%) | 5 (18%) |
| **現在従事されているお仕事の雇用形態は何ですか。** | | |
| 正社員・職員 | 17 (21%) | 2 (13%) |
| 契約社員・職員 | 5 (6.1%) | 1 (6.7%) |
| 派遣社員・職員 | 1 (1.2%) | 2 (13%) |
| パート・アルバイト | 46 (56%) | 9 (60%) |
| 自営・経営者 | 13 (16%) | 1 (6.7%) |
| 回答なし | 113 | 13 |
| **現在の世帯年収（税込み）はどのくらいですか。** | | |
| 0〜299万円 | 97 (50%) | 11 (39%) |

[1] Note: Items shown in this table were presented in Japanese in the original survey.

[2] Median (Q1, Q3); n (%)

| 質問項目[1] | 35歳以上<br>N = 195[2] | 35歳未満<br>N = 28[2] |
|---|---|---|
| 300〜599万円 | 69 (35%) | 11 (39%) |
| 600〜899万円 | 23 (12%) | 5 (18%) |
| 900〜1,199万円 | 4 (2.1%) | 1 (3.6%) |
| 1,200〜1,499万円 | 1 (0.5%) | 0 (0%) |
| 1,500万円以上 | 1 (0.5%) | 0 (0%) |

[1] Note: Items shown in this table were presented in Japanese in the original survey.

[2] Median (Q1, Q3); n (%)

**Table S4 Descriptive statistics of demographic characteristics and key study variables stratified by age in participants without schizophrenia**

| 質問項目[1] | 35歳以上 N = 1,291[2] | 35歳未満 N = 485[2] |
|---|---|---|
| **年齢** | 50 (43, 58) | 28 (24, 32) |
| **性別** | | |
| 男性 | 650 (50%) | 151 (31%) |
| 女性 | 641 (50%) | 334 (69%) |
| **あなたの現在の身長および体重をお答えください。（半角数字でご記入ください）※小数点以下は四捨五入してください。身長（cm）** | 164 (158, 171) | 161 (156, 168) |
| **あなたの現在の身長および体重をお答えください。（半角数字でご記入ください）※小数点以下は四捨五入してください。体重（kg）** | 60 (51, 70) | 53 (48, 62) |
| **現在の結婚状況についてお聞きします。1つだけ選択してください。** | | |
| 結婚・再婚・内縁（いずれも別居は除く） | 829 (64%) | 153 (32%) |
| 離婚 | 96 (7.4%) | 8 (1.6%) |
| 別居 | 10 (0.8%) | 0 (0%) |
| 死別 | 12 (0.9%) | 0 (0%) |
| 未婚 | 339 (26%) | 318 (66%) |
| その他 | 5 (0.4%) | 6 (1.2%) |
| **現在、どなたと一緒にお住まいですか。同居しているすべての人を選択してください。（いくつでも）　（同居・配偶者）** | | |
| なし | 473 (37%) | 335 (69%) |
| あり | 818 (63%) | 150 (31%) |
| **現在、どなたと一緒にお住まいですか。同居しているすべての人を選択してください。（いくつでも）　（同居・子供）** | | |
| なし | 786 (61%) | 385 (79%) |
| あり | 505 (39%) | 100 (21%) |
| **現在、どなたと一緒にお住まいですか。同居しているすべての人を選択してください。（いくつでも）　（同居・親）** | | |
| なし | 1,040 (81%) | 313 (65%) |
| あり | 251 (19%) | 172 (35%) |
| **現在、どなたと一緒にお住まいですか。同居しているすべての人を選択してください。（いくつでも）　（同居・その他）** | | |

[1] Note: Items shown in this table were presented in Japanese in the original survey.

[2] Median (Q1, Q3); n (%)

| 質問項目[1] | 35歳以上 N = 1,291[2] | 35歳未満 N = 485[2] |
|---|---|---|
| なし | 1,213 (94%) | 422 (87%) |
| あり | 78 (6.0%) | 63 (13%) |
| **現在、どなたと一緒にお住まいですか。同居しているすべての人を選択してください。（いくつでも） （同居・独り暮らし）** | | |
| なし | 1,072 (83%) | 336 (69%) |
| あり | 219 (17%) | 149 (31%) |
| **現在の同居人数は何人ですか。（半角数字でご記入ください）※あなたは含めません。** | | |
| 1 | 302 (28%) | 61 (18%) |
| 2 | 312 (29%) | 113 (34%) |
| 3 | 264 (25%) | 99 (29%) |
| 4 | 147 (14%) | 41 (12%) |
| 5 | 33 (3.1%) | 15 (4.5%) |
| 6 | 11 (1.0%) | 6 (1.8%) |
| 7 | 1 (<0.1%) | 1 (0.3%) |
| 8 | 2 (0.2%) | 0 (0%) |
| 回答なし | 219 | 149 |
| **現在の同居人数は何人ですか。（半角数字でご記入ください）※あなたは含めません。 （年齢14歳未満の同居人数）** | | |
| 0 | 216 (43%) | 0 (0%) |
| 1 | 157 (31%) | 65 (65%) |
| 2 | 107 (21%) | 29 (29%) |
| 3 | 23 (4.6%) | 6 (6.0%) |
| 4 | 2 (0.4%) | 0 (0%) |
| 回答なし | 786 | 385 |
| **全体的にみて、あなたの過去1か月間の健康状態はいかがでしたか。** | | |
| 最高に良い | 147 (11%) | 48 (9.9%) |
| やや良い | 294 (23%) | 138 (28%) |
| 良い | 645 (50%) | 226 (47%) |
| あまり良くない | 180 (14%) | 63 (13%) |
| 良くない | 25 (1.9%) | 10 (2.1%) |

[1] Note: Items shown in this table were presented in Japanese in the original survey.

[2] Median (Q1, Q3); n (%)

| 質問項目[1] | 35歳以上<br>N = 1,291[2] | 35歳未満<br>N = 485[2] |
|---|---|---|
| **現在の日常生活の状態はいかがですか。最も当てはまると思われるものを選んでください。** | | |
| 身体に特に障害はない | 1,207 (93%) | 465 (96%) |
| ＜身体に何らかの障害はあるが、日常生活はほぼ自分で出来、独力で外出する＞交通機関などを利用して外出する | 67 (5.2%) | 10 (2.1%) |
| ＜身体に何らかの障害はあるが、日常生活はほぼ自分で出来、独力で外出する＞隣近所にのみ出かける | 5 (0.4%) | 1 (0.2%) |
| ＜屋内での生活はおおむね自分で出来るが、介助なしには外出しない＞介助をしてもらって外出し、日中はほとんどベッドから離れて生活する | 1 (<0.1%) | 1 (0.2%) |
| ＜屋内での生活はおおむね自分で出来るが、介助なしには外出しない＞外出の頻度が少なく、日中も寝たり起きたりの生活をしている | 3 (0.2%) | 0 (0%) |
| ＜屋内での生活は何らかの介助を必要とし、日中もベッド上での生活が主であるが、座位を保つことができる＞車椅子に自分で乗り、食事、排泄はベッドから離れて行う | 1 (<0.1%) | 3 (0.6%) |
| ＜屋内での生活は何らかの介助を必要とし、日中もベッド上での生活が主であるが、座位を保つことができる＞介助により車椅子に乗る | 1 (<0.1%) | 0 (0%) |
| ＜1日中ベッド上で過ごし、排泄、食事、着替えの時に介助が要る＞自力で寝返りを打つ | 0 (0%) | 0 (0%) |
| ＜1日中ベッド上で過ごし、排泄、食事、着替えの時に介助が要る＞自力では寝返りも打たない | 6 (0.5%) | 5 (1.0%) |
| **昨年1年間の「身体の動かし方」についておたずねします。昨年1年間のうち、通常の時期の1日の時間の内訳を教えてください。通勤、仕事、家事などの時間をすべて含めてお答えください。余暇は含めません。: 1.座っている時間** | | |
| なかった | 33 (2.6%) | 22 (4.5%) |
| 1時間未満 | 28 (2.2%) | 15 (3.1%) |
| 1時間以上3時間未満 | 205 (16%) | 82 (17%) |
| 3時間以上5時間未満 | 353 (27%) | 138 (28%) |
| 5時間以上7時間未満 | 307 (24%) | 100 (21%) |
| 7時間以上9時間未満 | 174 (13%) | 77 (16%) |
| 9時間以上11時間未満 | 93 (7.2%) | 27 (5.6%) |
| 11時間以上 | 98 (7.6%) | 24 (4.9%) |
| **昨年1年間の「身体の動かし方」についておたずねします。昨年1年間のうち、通常の時期の1日の時間の内訳を教えてください。通勤、仕事、家事などの時間をすべて含めてお答えください。余暇は含めません。: 2.立っている時間** | | |
| なかった | 38 (2.9%) | 24 (4.9%) |
| 1時間未満 | 207 (16%) | 72 (15%) |
| 1時間以上3時間未満 | 469 (36%) | 169 (35%) |

[1] Note: Items shown in this table were presented in Japanese in the original survey.

[2] Median (Q1, Q3); n (%)

| 質問項目[1] | 35歳以上 N = 1,291[2] | 35歳未満 N = 485[2] |
|---|---|---|
| 3時間以上5時間未満 | 303 (23%) | 110 (23%) |
| 5時間以上7時間未満 | 154 (12%) | 58 (12%) |
| 7時間以上9時間未満 | 77 (6.0%) | 33 (6.8%) |
| 9時間以上11時間未満 | 26 (2.0%) | 12 (2.5%) |
| 11時間以上 | 17 (1.3%) | 7 (1.4%) |
| **昨年1年間の「身体の動かし方」についておたずねします。昨年1年間のうち、通常の時期の1日の時間の内訳を教えてください。通勤、仕事、家事などの時間をすべて含めてお答えください。余暇は含めません。: 3.歩いている時間** | | |
| なかった | 47 (3.6%) | 28 (5.8%) |
| 1時間未満 | 665 (52%) | 209 (43%) |
| 1時間以上3時間未満 | 456 (35%) | 178 (37%) |
| 3時間以上5時間未満 | 79 (6.1%) | 36 (7.4%) |
| 5時間以上7時間未満 | 23 (1.8%) | 18 (3.7%) |
| 7時間以上9時間未満 | 8 (0.6%) | 9 (1.9%) |
| 9時間以上11時間未満 | 6 (0.5%) | 3 (0.6%) |
| 11時間以上 | 7 (0.5%) | 4 (0.8%) |
| **昨年1年間の「身体の動かし方」についておたずねします。昨年1年間のうち、通常の時期の1日の時間の内訳を教えてください。通勤、仕事、家事などの時間をすべて含めてお答えください。余暇は含めません。: 4.力のいる作業をしている時間** | | |
| なかった | 503 (39%) | 166 (34%) |
| 1時間未満 | 584 (45%) | 216 (45%) |
| 1時間以上3時間未満 | 135 (10%) | 60 (12%) |
| 3時間以上5時間未満 | 39 (3.0%) | 24 (4.9%) |
| 5時間以上7時間未満 | 20 (1.5%) | 10 (2.1%) |
| 7時間以上9時間未満 | 6 (0.5%) | 9 (1.9%) |
| 9時間以上11時間未満 | 2 (0.2%) | 0 (0%) |
| 11時間以上 | 2 (0.2%) | 0 (0%) |
| **あなたの現在の状況について、以下の質問にお答えください。: 1.必要な時に、あなたの話を聞いてくれる人がいますか** | | |
| ほとんどいない | 152 (12%) | 47 (9.7%) |
| たまにいる | 224 (17%) | 79 (16%) |

[1] Note: Items shown in this table were presented in Japanese in the original survey.

[2] Median (Q1, Q3); n (%)

| 質問項目[1] | 35歳以上<br>N = 1,291[2] | 35歳未満<br>N = 485[2] |
|---|---|---|
| ときどきいる | 264 (20%) | 127 (26%) |
| よくいる | 314 (24%) | 133 (27%) |
| いつでもいる | 337 (26%) | 99 (20%) |
| **あなたの現在の状況について、以下の質問にお答えください。: 2.なにか困ったことがあった時、よいアドバイスをくれる人がいますか** | | |
| ほとんどいない | 201 (16%) | 59 (12%) |
| たまにいる | 261 (20%) | 94 (19%) |
| ときどきいる | 309 (24%) | 133 (27%) |
| よくいる | 269 (21%) | 114 (24%) |
| いつでもいる | 251 (19%) | 85 (18%) |
| **あなたの現在の状況について、以下の質問にお答えください。: 3.あなたを心配したり、あなたに愛情をかけてくれる人はいますか** | | |
| ほとんどいない | 136 (11%) | 36 (7.4%) |
| たまにいる | 218 (17%) | 85 (18%) |
| ときどきいる | 273 (21%) | 109 (22%) |
| よくいる | 289 (22%) | 109 (22%) |
| いつでもいる | 375 (29%) | 146 (30%) |
| **あなたの現在の状況について、以下の質問にお答えください。: 4.日常の家事をしたり、手伝ってくれる人はいますか** | | |
| ほとんどいない | 279 (22%) | 90 (19%) |
| たまにいる | 170 (13%) | 81 (17%) |
| ときどきいる | 250 (19%) | 104 (21%) |
| よくいる | 255 (20%) | 101 (21%) |
| いつでもいる | 337 (26%) | 109 (22%) |
| **あなたの現在の状況について、以下の質問にお答えください。: 5.あなたに情緒的な支えを与えてくれるような人、(たとえば、あなたの直面する問題について相談できる人、難しい判断が必要な時に助けてくれる人) はいますか** | | |
| ほとんどいない | 223 (17%) | 62 (13%) |
| たまにいる | 187 (14%) | 83 (17%) |

[1] Note: Items shown in this table were presented in Japanese in the original survey.

[2] Median (Q1, Q3); n (%)

| 質問項目[1] | 35歳以上<br>N = 1,291[2] | 35歳未満<br>N = 485[2] |
|---|---|---|
| ときどきいる | 271 (21%) | 113 (23%) |
| よくいる | 281 (22%) | 114 (24%) |
| いつでもいる | 329 (25%) | 113 (23%) |
| **あなたの現在の状況について、以下の質問にお答えください。: 6.必要な時にいつでも連絡がとれる、親しくて、信頼・信用できる人はいますか** | | |
| ほとんどいない | 197 (15%) | 55 (11%) |
| たまにいる | 207 (16%) | 70 (14%) |
| ときどきいる | 266 (21%) | 107 (22%) |
| よくいる | 287 (22%) | 125 (26%) |
| いつでもいる | 334 (26%) | 128 (26%) |
| **あなたの現在の状況について、以下の質問にお答えください。: 1.気軽に個人的な相談ができる親しい友人は何人いますか** | | |
| 0人 | 387 (30%) | 120 (25%) |
| 1人 | 271 (21%) | 102 (21%) |
| 2人 | 300 (23%) | 117 (24%) |
| 3人以上 | 333 (26%) | 146 (30%) |
| **あなたの現在の状況について、以下の質問にお答えください。: 2.気軽に個人的な相談ができる親類は何人いますか** | | |
| 0人 | 313 (24%) | 103 (21%) |
| 1人 | 349 (27%) | 138 (28%) |
| 2人 | 312 (24%) | 120 (25%) |
| 3人以上 | 317 (25%) | 124 (26%) |
| **地域組織、自助集団、チャリティー、ボランティアグループや、宗教団体などの集まりにどれくらいの頻度で参加していますか。** | | |
| 全く／ほとんど参加しない | 1,093 (85%) | 443 (91%) |
| 時々参加する | 145 (11%) | 26 (5.4%) |
| 週に1回未満 | 21 (1.6%) | 7 (1.4%) |
| 週に1回以上 | 32 (2.5%) | 9 (1.9%) |

[1] Note: Items shown in this table were presented in Japanese in the original survey.

[2] Median (Q1, Q3); n (%)

| 質問項目[1] | 35歳以上 N = 1,291[2] | 35歳未満 N = 485[2] |
|---|---|---|
| **あなたは、生きがいがあると感じていますか。** | | |
| 非常にある | 169 (13%) | 80 (16%) |
| ある | 639 (49%) | 240 (49%) |
| あまりない | 375 (29%) | 117 (24%) |
| 全くない | 108 (8.4%) | 48 (9.9%) |
| **あなたはご自分がどれくらい幸せだと感じていますか。** | | |
| 大変幸せ | 171 (13%) | 87 (18%) |
| 幸せ | 692 (54%) | 241 (50%) |
| どちらとも言えない | 333 (26%) | 117 (24%) |
| 幸せでない | 95 (7.4%) | 40 (8.2%) |
| **最近1週間の体や心の状態について、お聞きします。それぞれ最もあてはまる選択肢1つ選択してください。: 1.食べたくない。食欲がおちた** | | |
| 全く／ほとんどなかった | 1,065 (82%) | 375 (77%) |
| たまにあった（1〜2日） | 161 (12%) | 83 (17%) |
| しばしばあった（3〜4日） | 36 (2.8%) | 14 (2.9%) |
| いつもあった（ほぼ毎日） | 29 (2.2%) | 13 (2.7%) |
| **最近1週間の体や心の状態について、お聞きします。それぞれ最もあてはまる選択肢1つ選択してください。: 2.ゆううつだ** | | |
| 全く／ほとんどなかった | 799 (62%) | 236 (49%) |
| たまにあった（1〜2日） | 350 (27%) | 153 (32%) |
| しばしばあった（3〜4日） | 93 (7.2%) | 56 (12%) |
| いつもあった（ほぼ毎日） | 49 (3.8%) | 40 (8.2%) |
| **最近1週間の体や心の状態について、お聞きします。それぞれ最もあてはまる選択肢1つ選択してください。: 3.何をするのも面倒だ** | | |
| 全く／ほとんどなかった | 673 (52%) | 212 (44%) |
| たまにあった（1〜2日） | 455 (35%) | 157 (32%) |
| しばしばあった（3〜4日） | 108 (8.4%) | 65 (13%) |
| いつもあった（ほぼ毎日） | 55 (4.3%) | 51 (11%) |

[1] Note: Items shown in this table were presented in Japanese in the original survey.

[2] Median (Q1, Q3); n (%)

| 質問項目[1] | 35歳以上<br>N = 1,291[2] | 35歳未満<br>N = 485[2] |
|---|---|---|
| **最近1週間の体や心の状態について、お聞きします。それぞれ最もあてはまる選択肢1つ選択してください。: 4.なかなか眠れない** | | |
| 全く／ほとんどなかった | 846 (66%) | 274 (56%) |
| たまにあった（1〜2日） | 325 (25%) | 149 (31%) |
| しばしばあった（3〜4日） | 84 (6.5%) | 44 (9.1%) |
| いつもあった（ほぼ毎日） | 36 (2.8%) | 18 (3.7%) |
| **最近1週間の体や心の状態について、お聞きします。それぞれ最もあてはまる選択肢1つ選択してください。: 5.生活について満足して過ごせる** | | |
| 全く／ほとんどなかった | 303 (23%) | 147 (30%) |
| たまにあった（1〜2日） | 274 (21%) | 118 (24%) |
| しばしばあった（3〜4日） | 347 (27%) | 125 (26%) |
| いつもあった（ほぼ毎日） | 367 (28%) | 95 (20%) |
| **最近1週間の体や心の状態について、お聞きします。それぞれ最もあてはまる選択肢1つ選択してください。: 6.一人ぼっちでさびしい** | | |
| 全く／ほとんどなかった | 1,034 (80%) | 309 (64%) |
| たまにあった（1〜2日） | 185 (14%) | 104 (21%) |
| しばしばあった（3〜4日） | 40 (3.1%) | 47 (9.7%) |
| いつもあった（ほぼ毎日） | 32 (2.5%) | 25 (5.2%) |
| **最近1週間の体や心の状態について、お聞きします。それぞれ最もあてはまる選択肢1つ選択してください。: 7.皆がよそよそしいと思う** | | |
| 全く／ほとんどなかった | 1,053 (82%) | 371 (76%) |
| たまにあった（1〜2日） | 182 (14%) | 70 (14%) |
| しばしばあった（3〜4日） | 35 (2.7%) | 32 (6.6%) |
| いつもあった（ほぼ毎日） | 21 (1.6%) | 12 (2.5%) |
| **最近1週間の体や心の状態について、お聞きします。それぞれ最もあてはまる選択肢1つ選択してください。: 8.毎日が楽しい** | | |
| 全く／ほとんどなかった | 266 (21%) | 117 (24%) |
| たまにあった（1〜2日） | 409 (32%) | 161 (33%) |
| しばしばあった（3〜4日） | 364 (28%) | 131 (27%) |

[1] Note: Items shown in this table were presented in Japanese in the original survey.

[2] Median (Q1, Q3); n (%)

| 質問項目[1] | 35歳以上<br>N = 1,291[2] | 35歳未満<br>N = 485[2] |
|---|---|---|
| いつもあった（ほぼ毎日） | 252 (20%) | 76 (16%) |
| **最近1週間の体や心の状態について、お聞きします。それぞれ最もあてはまる選択肢1つ選択してください。: 9.悲しいと感じる** | | |
| 全く／ほとんどなかった | 829 (64%) | 266 (55%) |
| たまにあった（1〜2日） | 358 (28%) | 144 (30%) |
| しばしばあった（3〜4日） | 76 (5.9%) | 51 (11%) |
| いつもあった（ほぼ毎日） | 28 (2.2%) | 24 (4.9%) |
| **最近1週間の体や心の状態について、お聞きします。それぞれ最もあてはまる選択肢1つ選択してください。: 10.皆が自分を嫌っていると感じる** | | |
| 全く／ほとんどなかった | 1,048 (81%) | 350 (72%) |
| たまにあった（1〜2日） | 182 (14%) | 83 (17%) |
| しばしばあった（3〜4日） | 40 (3.1%) | 34 (7.0%) |
| いつもあった（ほぼ毎日） | 21 (1.6%) | 18 (3.7%) |
| **最近1週間の体や心の状態について、お聞きします。それぞれ最もあてはまる選択肢1つ選択してください。: 11.仕事が手につかない** | | |
| 全く／ほとんどなかった | 1,032 (80%) | 337 (69%) |
| たまにあった（1〜2日） | 205 (16%) | 99 (20%) |
| しばしばあった（3〜4日） | 45 (3.5%) | 32 (6.6%) |
| いつもあった（ほぼ毎日） | 9 (0.7%) | 17 (3.5%) |
| **あなたの気持ち、考えなどについてお伺いします。: 1.過去1か月、「人生での大切な事が自分の思うようにならない」と感じましたか** | | |
| 全くない | 363 (28%) | 137 (28%) |
| ほとんどない | 419 (32%) | 123 (25%) |
| ときどき | 388 (30%) | 166 (34%) |
| 頻繁に | 68 (5.3%) | 30 (6.2%) |
| とても頻繁に | 53 (4.1%) | 29 (6.0%) |
| **あなたの気持ち、考えなどについてお伺いします。: 2.過去1か月、「自分の問題を解決する能力に自信がある」と感じましたか** | | |
| 全くない | 203 (16%) | 115 (24%) |

[1] Note: Items shown in this table were presented in Japanese in the original survey.

[2] Median (Q1, Q3); n (%)

| 質問項目[1] | 35歳以上<br>N = 1,291[2] | 35歳未満<br>N = 485[2] |
|---|---|---|
| ほとんどない | 315 (24%) | 144 (30%) |
| ときどき | 524 (41%) | 175 (36%) |
| 頻繁に | 201 (16%) | 37 (7.6%) |
| とても頻繁に | 48 (3.7%) | 14 (2.9%) |
| **あなたの気持ち、考えなどについてお伺いします。: 3.過去1か月、「思うように物事がいっている」と感じましたか** | | |
| 全くない | 192 (15%) | 83 (17%) |
| ほとんどない | 289 (22%) | 107 (22%) |
| ときどき | 611 (47%) | 229 (47%) |
| 頻繁に | 170 (13%) | 49 (10%) |
| とても頻繁に | 29 (2.2%) | 17 (3.5%) |
| **あなたの気持ち、考えなどについてお伺いします。: 4.過去1か月、「多くの困難が山積みで自分の手に負えない」と感じましたか** | | |
| 全くない | 398 (31%) | 124 (26%) |
| ほとんどない | 486 (38%) | 159 (33%) |
| ときどき | 314 (24%) | 139 (29%) |
| 頻繁に | 63 (4.9%) | 39 (8.0%) |
| とても頻繁に | 30 (2.3%) | 24 (4.9%) |
| **あなたの周りの状況について、以下の質問にお答えください。: 1.一般的に、人は信用できると思いますか** | | |
| 全く思わない | 116 (9.0%) | 54 (11%) |
| あまり思わない | 471 (36%) | 196 (40%) |
| 思う | 658 (51%) | 212 (44%) |
| 非常によく思う | 46 (3.6%) | 23 (4.7%) |
| **あなたの周りの状況について、以下の質問にお答えください。: 2.多くの人は隙さえあれば、他の人を利用しようとするものだと思いますか** | | |
| 全く思わない | 106 (8.2%) | 40 (8.2%) |
| あまり思わない | 717 (56%) | 217 (45%) |
| 思う | 416 (32%) | 199 (41%) |

[1] Note: Items shown in this table were presented in Japanese in the original survey.

[2] Median (Q1, Q3); n (%)

| 質問項目[1] | 35歳以上 N = 1,291[2] | 35歳未満 N = 485[2] |
|---|---|---|
| 非常によく思う | 52 (4.0%) | 29 (6.0%) |
| **あなたの周りの状況について、以下の質問にお答えください。: 3.多くの場合、人は他の人の役に立とうとすると思いますか** | | |
| 全く思わない | 89 (6.9%) | 31 (6.4%) |
| あまり思わない | 490 (38%) | 195 (40%) |
| 思う | 684 (53%) | 240 (49%) |
| 非常によく思う | 28 (2.2%) | 19 (3.9%) |
| **学校教育はどのくらいまで受けられましたか。1つだけ選択してください。** | | |
| 中学校 | 25 (1.9%) | 7 (1.4%) |
| 高校 | 336 (26%) | 90 (19%) |
| 短大卒・専門学校・4年制大学中退 | 323 (25%) | 76 (16%) |
| 大学以上 | 597 (46%) | 309 (64%) |
| その他 | 10 (0.8%) | 3 (0.6%) |
| **現在の従事している職業は何ですか。** | | |
| 無職 | 125 (9.7%) | 60 (12%) |
| 主婦（主夫） | 216 (17%) | 46 (9.5%) |
| 専門的・技術的職業従事者 | 172 (13%) | 62 (13%) |
| 管理的職業従事者 | 100 (7.7%) | 1 (0.2%) |
| 事務従事者 | 223 (17%) | 115 (24%) |
| 販売従事者 | 60 (4.6%) | 22 (4.5%) |
| サービス職業従事者 | 130 (10%) | 50 (10%) |
| 保安職業従事者 | 7 (0.5%) | 5 (1.0%) |
| 農業漁業作業者 | 6 (0.5%) | 1 (0.2%) |
| 運輸・通信従事者 | 31 (2.4%) | 8 (1.6%) |
| 生産工程・労務作業者 | 66 (5.1%) | 16 (3.3%) |
| その他の職業 | 155 (12%) | 99 (20%) |
| **現在従事されているお仕事の雇用形態は何ですか。** | | |
| 正社員・職員 | 577 (61%) | 255 (67%) |

[1] Note: Items shown in this table were presented in Japanese in the original survey.

[2] Median (Q1, Q3); n (%)

| 質問項目[1] | 35歳以上<br>N = 1,291[2] | 35歳未満<br>N = 485[2] |
|---|---|---|
| 契約社員・職員 | 71 (7.5%) | 22 (5.8%) |
| 派遣社員・職員 | 34 (3.6%) | 10 (2.6%) |
| パート・アルバイト | 154 (16%) | 86 (23%) |
| 自営・経営者 | 114 (12%) | 6 (1.6%) |
| 回答なし | 341 | 106 |
| **現在の世帯年収（税込み）はどのくらいですか。** | | |
| 0〜299万円 | 239 (19%) | 120 (25%) |
| 300〜599万円 | 462 (36%) | 193 (40%) |
| 600〜899万円 | 325 (25%) | 103 (21%) |
| 900〜1,199万円 | 160 (12%) | 41 (8.5%) |
| 1,200〜1,499万円 | 65 (5.0%) | 10 (2.1%) |
| 1,500万円以上 | 40 (3.1%) | 18 (3.7%) |

[1] Note: Items shown in this table were presented in Japanese in the original survey.

[2] Median (Q1, Q3); n (%)

**Table S5 Descriptive statistics of demographic characteristics and key study variables stratified by sex in participants with schizophrenia**

| 質問項目[1] | 女性 N = 108[2] | 男性 N = 115[2] |
|---|---|---|
| **年齢** | 45 (36, 50) | 48 (42, 54) |
| **性別** | | |
| 男性 | 0 (0%) | 115 (100%) |
| 女性 | 108 (100%) | 0 (0%) |
| **あなたの現在の身長および体重をお答えください。（半角数字でご記入ください）※小数点以下は四捨五入してください。身長（cm）** | 158 (155, 163) | 171 (166, 174) |
| **あなたの現在の身長および体重をお答えください。（半角数字でご記入ください）※小数点以下は四捨五入してください。体重（kg）** | 60 (51, 70) | 75 (65, 88) |
| **現在の結婚状況についてお聞きします。1つだけ選択してください。** | | |
| 結婚・再婚・内縁（いずれも別居は除く） | 41 (38%) | 21 (18%) |
| 離婚 | 14 (13%) | 6 (5.2%) |
| 別居 | 3 (2.8%) | 0 (0%) |
| 死別 | 2 (1.9%) | 0 (0%) |
| 未婚 | 48 (44%) | 88 (77%) |
| その他 | 0 (0%) | 0 (0%) |
| **現在、どなたと一緒にお住まいですか。同居しているすべての人を選択してください。（いくつでも）（同居・配偶者）** | | |
| なし | 67 (62%) | 94 (82%) |
| あり | 41 (38%) | 21 (18%) |
| **現在、どなたと一緒にお住まいですか。同居しているすべての人を選択してください。（いくつでも）（同居・子供）** | | |
| なし | 76 (70%) | 100 (87%) |
| あり | 32 (30%) | 15 (13%) |
| **現在、どなたと一緒にお住まいですか。同居しているすべての人を選択してください。（いくつでも）（同居・親）** | | |
| なし | 58 (54%) | 42 (37%) |
| あり | 50 (46%) | 73 (63%) |
| **現在、どなたと一緒にお住まいですか。同居しているすべての人を選択してください。（いくつでも）（同居・その他）** | | |
| なし | 91 (84%) | 88 (77%) |
| あり | 17 (16%) | 27 (23%) |

[1] Note: Items shown in this table were presented in Japanese in the original survey.

[2] Median (Q1, Q3); n (%)

| 質問項目[1] | 女性 N = 108[2] | 男性 N = 115[2] |
|---|---|---|
| **現在、どなたと一緒にお住まいですか。同居しているすべての人を選択してください。（いくつでも）　（同居・独り暮らし）** | | |
| なし | 97 (90%) | 93 (81%) |
| あり | 11 (10%) | 22 (19%) |
| **現在の同居人数は何人ですか。（半角数字でご記入ください）※あなたは含めません。** | | |
| 1 | 31 (32%) | 21 (23%) |
| 2 | 34 (35%) | 39 (42%) |
| 3 | 23 (24%) | 20 (22%) |
| 4 | 6 (6.2%) | 9 (9.7%) |
| 5 | 2 (2.1%) | 3 (3.2%) |
| 6 | 1 (1.0%) | 0 (0%) |
| 8 | 0 (0%) | 1 (1.1%) |
| 回答なし | 11 | 22 |
| **現在の同居人数は何人ですか。（半角数字でご記入ください）※あなたは含めません。　（年齢14歳未満の同居人数）** | | |
| 0 | 12 (38%) | 8 (53%) |
| 1 | 14 (44%) | 3 (20%) |
| 2 | 6 (19%) | 4 (27%) |
| 回答なし | 76 | 100 |
| **全体的にみて、あなたの過去1か月間の健康状態はいかがでしたか。** | | |
| 最高に良い | 1 (0.9%) | 5 (4.3%) |
| やや良い | 9 (8.3%) | 24 (21%) |
| 良い | 41 (38%) | 35 (30%) |
| あまり良くない | 42 (39%) | 42 (37%) |
| 良くない | 15 (14%) | 9 (7.8%) |
| **現在の日常生活の状態はいかがですか。最も当てはまると思われるものを選んでください。** | | |
| 身体に特に障害はない | 64 (59%) | 70 (61%) |
| ＜身体に何らかの障害はあるが、日常生活はほぼ自分で出来、独力で外出する＞交通機関などを利用して外出する | 21 (19%) | 15 (13%) |
| ＜身体に何らかの障害はあるが、日常生活はほぼ自分で出来、独力で外出する＞隣近所にのみ出かける | 17 (16%) | 22 (19%) |
| ＜屋内での生活はおおむね自分で出来るが、介助なしには外出しない＞介助をしてもらって外出し、日中はほとんどベッドから離れて生活する | 3 (2.8%) | 2 (1.7%) |
| ＜屋内での生活はおおむね自分で出来るが、介助なしには外出しない＞外出の頻度が少なく、日中も寝たり起きたりの生活をしている | 1 (0.9%) | 4 (3.5%) |

[1] Note: Items shown in this table were presented in Japanese in the original survey.

[2] Median (Q1, Q3); n (%)

| **質問項目**[1] | **女性**<br>N = 108[2] | **男性**<br>N = 115[2] |
|---|---|---|
| ＜屋内での生活は何らかの介助を必要とし、日中もベッド上での生活が主であるが、座位を保つことができる＞車椅子に自分で乗り、食事、排泄はベッドから離れて行う | 2 (1.9%) | 1 (0.9%) |
| ＜屋内での生活は何らかの介助を必要とし、日中もベッド上での生活が主であるが、座位を保つことができる＞介助により車椅子に乗る | 0 (0%) | 1 (0.9%) |
| ＜1日中ベッド上で過ごし、排泄、食事、着替えの時に介助が要る＞自力で寝返りを打つ | 0 (0%) | 0 (0%) |
| ＜1日中ベッド上で過ごし、排泄、食事、着替えの時に介助が要る＞自力では寝返りも打たない | 0 (0%) | 0 (0%) |
| **昨年1年間の「身体の動かし方」についておたずねします。昨年1年間のうち、通常の時期の1日の時間の内訳を教えてください。通勤、仕事、家事などの時間をすべて含めてお答えください。余暇は含めません。：1.座っている時間** | | |
| なかった | 0 (0%) | 0 (0%) |
| 1時間未満 | 7 (6.5%) | 9 (7.8%) |
| 1時間以上3時間未満 | 16 (15%) | 25 (22%) |
| 3時間以上5時間未満 | 30 (28%) | 25 (22%) |
| 5時間以上7時間未満 | 20 (19%) | 25 (22%) |
| 7時間以上9時間未満 | 18 (17%) | 10 (8.7%) |
| 9時間以上11時間未満 | 8 (7.4%) | 7 (6.1%) |
| 11時間以上 | 9 (8.3%) | 14 (12%) |
| **昨年1年間の「身体の動かし方」についておたずねします。昨年1年間のうち、通常の時期の1日の時間の内訳を教えてください。通勤、仕事、家事などの時間をすべて含めてお答えください。余暇は含めません。：2.立っている時間** | | |
| なかった | 2 (1.9%) | 6 (5.2%) |
| 1時間未満 | 29 (27%) | 37 (32%) |
| 1時間以上3時間未満 | 47 (44%) | 43 (37%) |
| 3時間以上5時間未満 | 21 (19%) | 13 (11%) |
| 5時間以上7時間未満 | 2 (1.9%) | 8 (7.0%) |
| 7時間以上9時間未満 | 4 (3.7%) | 4 (3.5%) |
| 9時間以上11時間未満 | 0 (0%) | 3 (2.6%) |
| 11時間以上 | 3 (2.8%) | 1 (0.9%) |
| **昨年1年間の「身体の動かし方」についておたずねします。昨年1年間のうち、通常の時期の1日の時間の内訳を教えてください。通勤、仕事、家事などの時間をすべて含めてお答えください。余暇は含めません。：3.歩いている時間** | | |
| なかった | 3 (2.8%) | 7 (6.1%) |
| 1時間未満 | 65 (60%) | 59 (51%) |
| 1時間以上3時間未満 | 34 (31%) | 44 (38%) |

[1] Note: Items shown in this table were presented in Japanese in the original survey.

[2] Median (Q1, Q3); n (%)

| 質問項目[1] | 女性<br>N = 108[2] | 男性<br>N = 115[2] |
|---|---|---|
| 3時間以上5時間未満 | 5 (4.6%) | 3 (2.6%) |
| 5時間以上7時間未満 | 0 (0%) | 0 (0%) |
| 7時間以上9時間未満 | 0 (0%) | 1 (0.9%) |
| 9時間以上11時間未満 | 0 (0%) | 1 (0.9%) |
| 11時間以上 | 1 (0.9%) | 0 (0%) |
| **昨年1年間の「身体の動かし方」についておたずねします。昨年1年間のうち、通常の時期の1日の時間の内訳を教えてください。通勤、仕事、家事などの時間をすべて含めてお答えください。余暇は含めません。: 4.力のいる作業をしている時間** | | |
| なかった | 55 (51%) | 61 (53%) |
| 1時間未満 | 42 (39%) | 36 (31%) |
| 1時間以上3時間未満 | 5 (4.6%) | 10 (8.7%) |
| 3時間以上5時間未満 | 4 (3.7%) | 4 (3.5%) |
| 5時間以上7時間未満 | 1 (0.9%) | 0 (0%) |
| 7時間以上9時間未満 | 0 (0%) | 2 (1.7%) |
| 9時間以上11時間未満 | 0 (0%) | 1 (0.9%) |
| 11時間以上 | 1 (0.9%) | 1 (0.9%) |
| **あなたの現在の状況について、以下の質問にお答えください。: 1.必要な時に、あなたの話を聞いてくれる人がいますか** | | |
| ほとんどいない | 15 (14%) | 19 (17%) |
| たまにいる | 26 (24%) | 23 (20%) |
| ときどきいる | 21 (19%) | 27 (23%) |
| よくいる | 28 (26%) | 19 (17%) |
| いつでもいる | 18 (17%) | 27 (23%) |
| **あなたの現在の状況について、以下の質問にお答えください。: 2.なにか困ったことがあった時、よいアドバイスをくれる人がいますか** | | |
| ほとんどいない | 16 (15%) | 24 (21%) |
| たまにいる | 28 (26%) | 25 (22%) |
| ときどきいる | 18 (17%) | 23 (20%) |
| よくいる | 32 (30%) | 22 (19%) |
| いつでもいる | 14 (13%) | 21 (18%) |
| **あなたの現在の状況について、以下の質問にお答えください。: 3.あなたを心配したり、あなたに愛情をかけてくれる人はいますか** | | |
| ほとんどいない | 10 (9.3%) | 17 (15%) |

[1] Note: Items shown in this table were presented in Japanese in the original survey.

[2] Median (Q1, Q3); n (%)

| 質問項目[1] | 女性<br>N = 108[2] | 男性<br>N = 115[2] |
|---|---|---|
| たまにいる | 19 (18%) | 20 (17%) |
| ときどきいる | 25 (23%) | 31 (27%) |
| よくいる | 24 (22%) | 19 (17%) |
| いつでもいる | 30 (28%) | 28 (24%) |
| **あなたの現在の状況について、以下の質問にお答えください。: 4.日常の家事をしたり、手伝ってくれる人はいますか** | | |
| ほとんどいない | 23 (21%) | 20 (17%) |
| たまにいる | 22 (20%) | 19 (17%) |
| ときどきいる | 13 (12%) | 12 (10%) |
| よくいる | 22 (20%) | 28 (24%) |
| いつでもいる | 28 (26%) | 36 (31%) |
| **あなたの現在の状況について、以下の質問にお答えください。: 5.あなたに情緒的な支えを与えてくれるような人、（たとえば、あなたの直面する問題について相談できる人、難しい判断が必要な時に助けてくれる人）はいますか** | | |
| ほとんどいない | 19 (18%) | 24 (21%) |
| たまにいる | 21 (19%) | 23 (20%) |
| ときどきいる | 22 (20%) | 23 (20%) |
| よくいる | 23 (21%) | 20 (17%) |
| いつでもいる | 23 (21%) | 25 (22%) |
| **あなたの現在の状況について、以下の質問にお答えください。: 6.必要な時にいつでも連絡がとれる、親しくて、信頼・信用できる人はいますか** | | |
| ほとんどいない | 24 (22%) | 23 (20%) |
| たまにいる | 18 (17%) | 22 (19%) |
| ときどきいる | 16 (15%) | 25 (22%) |
| よくいる | 24 (22%) | 19 (17%) |
| いつでもいる | 26 (24%) | 26 (23%) |
| **あなたの現在の状況について、以下の質問にお答えください。: 1.気軽に個人的な相談ができる親しい友人は何人いますか** | | |
| 0人 | 36 (33%) | 56 (49%) |
| 1人 | 36 (33%) | 25 (22%) |
| 2人 | 21 (19%) | 17 (15%) |
| 3人以上 | 15 (14%) | 17 (15%) |

[1] Note: Items shown in this table were presented in Japanese in the original survey.

[2] Median (Q1, Q3); n (%)

| 質問項目[1] | 女性<br>N = 108[2] | 男性<br>N = 115[2] |
|---|---|---|
| **あなたの現在の状況について、以下の質問にお答えください。: 2.気軽に個人的な相談ができる親類は何人いますか** | | |
| 0人 | 37 (34%) | 36 (31%) |
| 1人 | 35 (32%) | 32 (28%) |
| 2人 | 16 (15%) | 24 (21%) |
| 3人以上 | 20 (19%) | 23 (20%) |
| **地域組織、自助集団、チャリティー、ボランティアグループや、宗教団体などの集まりにどれくらいの頻度で参加していますか。** | | |
| 全く／ほとんど参加しない | 90 (83%) | 99 (86%) |
| 時々参加する | 17 (16%) | 5 (4.3%) |
| 週に1回未満 | 1 (0.9%) | 4 (3.5%) |
| 週に1回以上 | 0 (0%) | 7 (6.1%) |
| **あなたは、生きがいがあると感じていますか。** | | |
| 非常にある | 10 (9.3%) | 14 (12%) |
| ある | 38 (35%) | 35 (30%) |
| あまりない | 43 (40%) | 46 (40%) |
| 全くない | 17 (16%) | 20 (17%) |
| **あなたはご自分がどれくらい幸せだと感じていますか。** | | |
| 大変幸せ | 12 (11%) | 10 (8.7%) |
| 幸せ | 44 (41%) | 32 (28%) |
| どちらとも言えない | 29 (27%) | 43 (37%) |
| 幸せでない | 23 (21%) | 30 (26%) |
| **最近1週間の体や心の状態について、お聞きします。それぞれ最もあてはまる選択肢1つ選択してください。: 1.食べたくない。食欲がおちた** | | |
| 全く／ほとんどなかった | 63 (58%) | 87 (76%) |
| たまにあった（1〜2日） | 28 (26%) | 19 (17%) |
| しばしばあった（3〜4日） | 7 (6.5%) | 5 (4.3%) |
| いつもあった（ほぼ毎日） | 10 (9.3%) | 4 (3.5%) |
| **最近1週間の体や心の状態について、お聞きします。それぞれ最もあてはまる選択肢1つ選択してください。: 2.ゆううつだ** | | |
| 全く／ほとんどなかった | 22 (20%) | 29 (25%) |
| たまにあった（1〜2日） | 41 (38%) | 43 (37%) |
| しばしばあった（3〜4日） | 26 (24%) | 19 (17%) |

[1] Note: Items shown in this table were presented in Japanese in the original survey.

[2] Median (Q1, Q3); n (%)

| 質問項目[1] | 女性<br>N = 108[2] | 男性<br>N = 115[2] |
|---|---|---|
| いつもあった（ほぼ毎日） | 19 (18%) | 24 (21%) |
| **最近1週間の体や心の状態について、お聞きします。それぞれ最もあてはまる選択肢1つ選択してください。: 3.何をするのも面倒だ** | | |
| 全く／ほとんどなかった | 14 (13%) | 25 (22%) |
| たまにあった（1〜2日） | 41 (38%) | 45 (39%) |
| しばしばあった（3〜4日） | 27 (25%) | 18 (16%) |
| いつもあった（ほぼ毎日） | 26 (24%) | 27 (23%) |
| **最近1週間の体や心の状態について、お聞きします。それぞれ最もあてはまる選択肢1つ選択してください。: 4.なかなか眠れない** | | |
| 全く／ほとんどなかった | 30 (28%) | 46 (40%) |
| たまにあった（1〜2日） | 37 (34%) | 28 (24%) |
| しばしばあった（3〜4日） | 22 (20%) | 15 (13%) |
| いつもあった（ほぼ毎日） | 19 (18%) | 26 (23%) |
| **最近1週間の体や心の状態について、お聞きします。それぞれ最もあてはまる選択肢1つ選択してください。: 5.生活について満足して過ごせる** | | |
| 全く／ほとんどなかった | 24 (22%) | 34 (30%) |
| たまにあった（1〜2日） | 33 (31%) | 35 (30%) |
| しばしばあった（3〜4日） | 36 (33%) | 23 (20%) |
| いつもあった（ほぼ毎日） | 15 (14%) | 23 (20%) |
| **最近1週間の体や心の状態について、お聞きします。それぞれ最もあてはまる選択肢1つ選択してください。: 6.一人ぼっちでさびしい** | | |
| 全く／ほとんどなかった | 55 (51%) | 64 (56%) |
| たまにあった（1〜2日） | 33 (31%) | 31 (27%) |
| しばしばあった（3〜4日） | 12 (11%) | 12 (10%) |
| いつもあった（ほぼ毎日） | 8 (7.4%) | 8 (7.0%) |
| **最近1週間の体や心の状態について、お聞きします。それぞれ最もあてはまる選択肢1つ選択してください。: 7.皆がよそよそしいと思う** | | |
| 全く／ほとんどなかった | 59 (55%) | 66 (57%) |
| たまにあった（1〜2日） | 33 (31%) | 27 (23%) |
| しばしばあった（3〜4日） | 8 (7.4%) | 14 (12%) |
| いつもあった（ほぼ毎日） | 8 (7.4%) | 8 (7.0%) |
| **最近1週間の体や心の状態について、お聞きします。それぞれ最もあてはまる選択肢1つ選択してください。: 8.毎日が楽しい** | | |

[1] Note: Items shown in this table were presented in Japanese in the original survey.

[2] Median (Q1, Q3); n (%)

| 質問項目[1] | 女性<br>N = 108[2] | 男性<br>N = 115[2] |
|---|---|---|
| 全く／ほとんどなかった | 30 (28%) | 41 (36%) |
| たまにあった（1〜2日） | 39 (36%) | 45 (39%) |
| しばしばあった（3〜4日） | 28 (26%) | 18 (16%) |
| いつもあった（ほぼ毎日） | 11 (10%) | 11 (9.6%) |
| **最近1週間の体や心の状態について、お聞きします。それぞれ最もあてはまる選択肢1つ選択してください。: 9.悲しいと感じる** | | |
| 全く／ほとんどなかった | 23 (21%) | 43 (37%) |
| たまにあった（1〜2日） | 45 (42%) | 41 (36%) |
| しばしばあった（3〜4日） | 24 (22%) | 20 (17%) |
| いつもあった（ほぼ毎日） | 16 (15%) | 11 (9.6%) |
| **最近1週間の体や心の状態について、お聞きします。それぞれ最もあてはまる選択肢1つ選択してください。: 10.皆が自分を嫌っていると感じる** | | |
| 全く／ほとんどなかった | 41 (38%) | 55 (48%) |
| たまにあった（1〜2日） | 39 (36%) | 29 (25%) |
| しばしばあった（3〜4日） | 14 (13%) | 14 (12%) |
| いつもあった（ほぼ毎日） | 14 (13%) | 17 (15%) |
| **最近1週間の体や心の状態について、お聞きします。それぞれ最もあてはまる選択肢1つ選択してください。: 11.仕事が手につかない** | | |
| 全く／ほとんどなかった | 47 (44%) | 54 (47%) |
| たまにあった（1〜2日） | 33 (31%) | 31 (27%) |
| しばしばあった（3〜4日） | 14 (13%) | 10 (8.7%) |
| いつもあった（ほぼ毎日） | 14 (13%) | 20 (17%) |
| **あなたの気持ち、考えなどについてお伺いします。: 1.過去1か月、「人生での大切な事が自分の思うようにならない」と感じましたか** | | |
| 全くない | 4 (3.7%) | 10 (8.7%) |
| ほとんどない | 14 (13%) | 23 (20%) |
| ときどき | 52 (48%) | 37 (32%) |
| 頻繁に | 18 (17%) | 22 (19%) |
| とても頻繁に | 20 (19%) | 23 (20%) |
| **あなたの気持ち、考えなどについてお伺いします。: 2.過去1か月、「自分の問題を解決する能力に自信がある」と感じましたか** | | |
| 全くない | 35 (32%) | 31 (27%) |
| ほとんどない | 35 (32%) | 37 (32%) |

[1] Note: Items shown in this table were presented in Japanese in the original survey.

[2] Median (Q1, Q3); n (%)

| 質問項目[1] | 女性<br>N = 108[2] | 男性<br>N = 115[2] |
|---|---|---|
| ときどき | 27 (25%) | 38 (33%) |
| 頻繁に | 10 (9.3%) | 8 (7.0%) |
| とても頻繁に | 1 (0.9%) | 1 (0.9%) |
| **あなたの気持ち、考えなどについてお伺いします。: 3.過去1か月、「思うように物事がいっている」と感じましたか** | | |
| 全くない | 26 (24%) | 26 (23%) |
| ほとんどない | 32 (30%) | 43 (37%) |
| ときどき | 43 (40%) | 35 (30%) |
| 頻繁に | 5 (4.6%) | 9 (7.8%) |
| とても頻繁に | 2 (1.9%) | 2 (1.7%) |
| **あなたの気持ち、考えなどについてお伺いします。: 4.過去1か月、「多くの困難が山積みで自分の手に負えない」と感じましたか** | | |
| 全くない | 9 (8.3%) | 13 (11%) |
| ほとんどない | 31 (29%) | 35 (30%) |
| ときどき | 33 (31%) | 36 (31%) |
| 頻繁に | 19 (18%) | 16 (14%) |
| とても頻繁に | 16 (15%) | 15 (13%) |
| **あなたの周りの状況について、以下の質問にお答えください。: 1.一般的に、人は信用できると思いますか** | | |
| 全く思わない | 18 (17%) | 19 (17%) |
| あまり思わない | 49 (45%) | 55 (48%) |
| 思う | 37 (34%) | 38 (33%) |
| 非常によく思う | 4 (3.7%) | 3 (2.6%) |
| **あなたの周りの状況について、以下の質問にお答えください。: 2.多くの人は隙さえあれば、他の人を利用しようとするものだと思いますか** | | |
| 全く思わない | 1 (0.9%) | 8 (7.0%) |
| あまり思わない | 49 (45%) | 48 (42%) |
| 思う | 49 (45%) | 46 (40%) |
| 非常によく思う | 9 (8.3%) | 13 (11%) |
| **あなたの周りの状況について、以下の質問にお答えください。: 3.多くの場合、人は他の人の役に立とうとすると思いますか** | | |
| 全く思わない | 6 (5.6%) | 11 (9.6%) |
| あまり思わない | 44 (41%) | 50 (43%) |
| 思う | 55 (51%) | 48 (42%) |

[1] Note: Items shown in this table were presented in Japanese in the original survey.

[2] Median (Q1, Q3); n (%)

| 質問項目[1] | 女性<br>N = 108[2] | 男性<br>N = 115[2] |
|---|---|---|
| 非常によく思う | 3 (2.8%) | 6 (5.2%) |
| **学校教育はどのくらいまで受けられましたか。1つだけ選択してください。** | | |
| 中学校 | 6 (5.6%) | 7 (6.1%) |
| 高校 | 35 (32%) | 43 (37%) |
| 短大卒・専門学校・4年制大学中退 | 34 (31%) | 17 (15%) |
| 大学以上 | 31 (29%) | 47 (41%) |
| その他 | 2 (1.9%) | 1 (0.9%) |
| **現在の従事している職業は何ですか。** | | |
| 無職 | 36 (33%) | 58 (50%) |
| 主婦（主夫） | 29 (27%) | 3 (2.6%) |
| 専門的・技術的職業従事者 | 4 (3.7%) | 5 (4.3%) |
| 管理的職業従事者 | 0 (0%) | 3 (2.6%) |
| 事務従事者 | 10 (9.3%) | 7 (6.1%) |
| 販売従事者 | 7 (6.5%) | 1 (0.9%) |
| サービス職業従事者 | 4 (3.7%) | 5 (4.3%) |
| 保安職業従事者 | 0 (0%) | 0 (0%) |
| 農業漁業作業者 | 0 (0%) | 2 (1.7%) |
| 運輸・通信従事者 | 0 (0%) | 2 (1.7%) |
| 生産工程・労務作業者 | 6 (5.6%) | 10 (8.7%) |
| その他の職業 | 12 (11%) | 19 (17%) |
| **現在従事されているお仕事の雇用形態は何ですか。** | | |
| 正社員・職員 | 5 (12%) | 14 (26%) |
| 契約社員・職員 | 2 (4.7%) | 4 (7.4%) |
| 派遣社員・職員 | 3 (7.0%) | 0 (0%) |
| パート・アルバイト | 29 (67%) | 26 (48%) |
| 自営・経営者 | 4 (9.3%) | 10 (19%) |
| 回答なし | 65 | 61 |
| **現在の世帯年収（税込み）はどのくらいですか。** | | |
| 0〜299万円 | 47 (44%) | 61 (53%) |
| 300〜599万円 | 40 (37%) | 40 (35%) |
| 600〜899万円 | 16 (15%) | 12 (10%) |

[1] Note: Items shown in this table were presented in Japanese in the original survey.

[2] Median (Q1, Q3); n (%)

| 質問項目[1] | 女性<br>N = 108[2] | 男性<br>N = 115[2] |
|---|---|---|
| 900〜1,199万円 | 4 (3.7%) | 1 (0.9%) |
| 1,200〜1,499万円 | 1 (0.9%) | 0 (0%) |
| 1,500万円以上 | 0 (0%) | 1 (0.9%) |

[1] Note: Items shown in this table were presented in Japanese in the original survey.

[2] Median (Q1, Q3); n (%)

**Table S6 Descriptive statistics of demographic characteristics and key study variables stratified by sex in participants without schizophrenia**

| 質問項目[1] | 女性 N = 975[2] | 男性 N = 801[2] |
|---|---|---|
| **年齢** | 41 (31, 51) | 49 (38, 58) |
| **性別** | | |
| 男性 | 0 (0%) | 801 (100%) |
| 女性 | 975 (100%) | 0 (0%) |
| **あなたの現在の身長および体重をお答えください。（半角数字でご記入ください）※小数点以下は四捨五入してください。身長（cm）** | 158 (154, 162) | 171 (167, 175) |
| **あなたの現在の身長および体重をお答えください。（半角数字でご記入ください）※小数点以下は四捨五入してください。体重（kg）** | 51 (47, 56) | 68 (60, 75) |
| **現在の結婚状況についてお聞きします。1つだけ選択してください。** | | |
| 結婚・再婚・内縁（いずれも別居は除く） | 519 (53%) | 463 (58%) |
| 離婚 | 67 (6.9%) | 37 (4.6%) |
| 別居 | 4 (0.4%) | 6 (0.7%) |
| 死別 | 10 (1.0%) | 2 (0.2%) |
| 未婚 | 371 (38%) | 286 (36%) |
| その他 | 4 (0.4%) | 7 (0.9%) |
| **現在、どなたと一緒にお住まいですか。同居しているすべての人を選択してください。（いくつでも）（同居・配偶者）** | | |
| なし | 463 (47%) | 345 (43%) |
| あり | 512 (53%) | 456 (57%) |
| **現在、どなたと一緒にお住まいですか。同居しているすべての人を選択してください。（いくつでも）（同居・子供）** | | |
| なし | 627 (64%) | 544 (68%) |
| あり | 348 (36%) | 257 (32%) |
| **現在、どなたと一緒にお住まいですか。同居しているすべての人を選択してください。（いくつでも）（同居・親）** | | |
| なし | 746 (77%) | 607 (76%) |
| あり | 229 (23%) | 194 (24%) |

[1] Note: Items shown in this table were presented in Japanese in the original survey.

[2] Median (Q1, Q3); n (%)

| 質問項目[1] | 女性<br>N = 975[2] | 男性<br>N = 801[2] |
|---|---|---|
| **現在、どなたと一緒にお住まいですか。同居しているすべての人を選択してください。（いくつでも）　（同居・その他）** | | |
| なし | 884 (91%) | 751 (94%) |
| あり | 91 (9.3%) | 50 (6.2%) |
| **現在、どなたと一緒にお住まいですか。同居しているすべての人を選択してください。（いくつでも）　（同居・独り暮らし）** | | |
| なし | 784 (80%) | 624 (78%) |
| あり | 191 (20%) | 177 (22%) |
| **現在の同居人数は何人ですか。（半角数字でご記入ください）※あなたは含めません。** | | |
| 1 | 200 (26%) | 163 (26%) |
| 2 | 247 (32%) | 178 (29%) |
| 3 | 201 (26%) | 162 (26%) |
| 4 | 105 (13%) | 83 (13%) |
| 5 | 23 (2.9%) | 25 (4.0%) |
| 6 | 7 (0.9%) | 10 (1.6%) |
| 7 | 1 (0.1%) | 1 (0.2%) |
| 8 | 0 (0%) | 2 (0.3%) |
| 回答なし | 191 | 177 |
| **現在の同居人数は何人ですか。（半角数字でご記入ください）※あなたは含めません。　（年齢14歳未満の同居人数）** | | |
| 0 | 108 (31%) | 108 (42%) |
| 1 | 141 (41%) | 81 (32%) |
| 2 | 77 (22%) | 59 (23%) |
| 3 | 21 (6.0%) | 8 (3.1%) |
| 4 | 1 (0.3%) | 1 (0.4%) |
| 回答なし | 627 | 544 |
| **全体的にみて、あなたの過去1か月間の健康状態はいかがでしたか。** | | |
| 最高に良い | 103 (11%) | 92 (11%) |
| やや良い | 256 (26%) | 176 (22%) |
| 良い | 474 (49%) | 397 (50%) |

[1] Note: Items shown in this table were presented in Japanese in the original survey.

[2] Median (Q1, Q3); n (%)

| 質問項目[1] | 女性<br>N = 975[2] | 男性<br>N = 801[2] |
|---|---|---|
| あまり良くない | 127 (13%) | 116 (14%) |
| 良くない | 15 (1.5%) | 20 (2.5%) |
| **現在の日常生活の状態はいかがですか。最も当てはまると思われるものを選んでください。** | | |
| 身体に特に障害はない | 930 (95%) | 742 (93%) |
| ＜身体に何らかの障害はあるが、日常生活はほぼ自分で出来、独力で外出する＞交通機関などを利用して外出する | 29 (3.0%) | 48 (6.0%) |
| ＜身体に何らかの障害はあるが、日常生活はほぼ自分で出来、独力で外出する＞隣近所にのみ出かける | 4 (0.4%) | 2 (0.2%) |
| ＜屋内での生活はおおむね自分で出来るが、介助なしには外出しない＞介助をしてもらって外出し、日中はほとんどベッドから離れて生活する | 1 (0.1%) | 1 (0.1%) |
| ＜屋内での生活はおおむね自分で出来るが、介助なしには外出しない＞外出の頻度が少なく、日中も寝たり起きたりの生活をしている | 2 (0.2%) | 1 (0.1%) |
| ＜屋内での生活は何らかの介助を必要とし、日中もベッド上での生活が主であるが、座位を保つことができる＞車椅子に自分で乗り、食事、排泄はベッドから離れて行う | 3 (0.3%) | 1 (0.1%) |
| ＜屋内での生活は何らかの介助を必要とし、日中もベッド上での生活が主であるが、座位を保つことができる＞介助により車椅子に乗る | 1 (0.1%) | 0 (0%) |
| ＜1日中ベッド上で過ごし、排泄、食事、着替えの時に介助が要る＞自力で寝返りを打つ | 0 (0%) | 0 (0%) |
| ＜1日中ベッド上で過ごし、排泄、食事、着替えの時に介助が要る＞自力では寝返りも打たない | 5 (0.5%) | 6 (0.7%) |
| **昨年1年間の「身体の動かし方」についておたずねします。昨年1年間のうち、通常の時期の1日の時間の内訳を教えてください。通勤、仕事、家事などの時間をすべて含めてお答えください。余暇は含めません。: 1.座っている時間** | | |
| なかった | 32 (3.3%) | 23 (2.9%) |
| 1時間未満 | 23 (2.4%) | 20 (2.5%) |
| 1時間以上3時間未満 | 160 (16%) | 127 (16%) |
| 3時間以上5時間未満 | 283 (29%) | 208 (26%) |
| 5時間以上7時間未満 | 207 (21%) | 200 (25%) |
| 7時間以上9時間未満 | 146 (15%) | 105 (13%) |
| 9時間以上11時間未満 | 70 (7.2%) | 50 (6.2%) |
| 11時間以上 | 54 (5.5%) | 68 (8.5%) |
| **昨年1年間の「身体の動かし方」についておたずねします。昨年1年間のうち、通常の時期の1日の時間の内訳を教えてください。通勤、仕事、家事などの時間をすべて含めてお答えください。余暇は含めません。: 2.立っている時間** | | |

[1] Note: Items shown in this table were presented in Japanese in the original survey.

[2] Median (Q1, Q3); n (%)

| 質問項目[1] | 女性<br>N = 975[2] | 男性<br>N = 801[2] |
|---|---|---|
| なかった | 33 (3.4%) | 29 (3.6%) |
| 1時間未満 | 122 (13%) | 157 (20%) |
| 1時間以上3時間未満 | 334 (34%) | 304 (38%) |
| 3時間以上5時間未満 | 244 (25%) | 169 (21%) |
| 5時間以上7時間未満 | 136 (14%) | 76 (9.5%) |
| 7時間以上9時間未満 | 72 (7.4%) | 38 (4.7%) |
| 9時間以上11時間未満 | 21 (2.2%) | 17 (2.1%) |
| 11時間以上 | 13 (1.3%) | 11 (1.4%) |
| **昨年1年間の「身体の動かし方」についておたずねします。昨年1年間のうち、通常の時期の1日の時間の内訳を教えてください。通勤、仕事、家事などの時間をすべて含めてお答えください。余暇は含めません。: 3.歩いている時間** | | |
| なかった | 43 (4.4%) | 32 (4.0%) |
| 1時間未満 | 464 (48%) | 410 (51%) |
| 1時間以上3時間未満 | 348 (36%) | 286 (36%) |
| 3時間以上5時間未満 | 64 (6.6%) | 51 (6.4%) |
| 5時間以上7時間未満 | 33 (3.4%) | 8 (1.0%) |
| 7時間以上9時間未満 | 12 (1.2%) | 5 (0.6%) |
| 9時間以上11時間未満 | 4 (0.4%) | 5 (0.6%) |
| 11時間以上 | 7 (0.7%) | 4 (0.5%) |
| **昨年1年間の「身体の動かし方」についておたずねします。昨年1年間のうち、通常の時期の1日の時間の内訳を教えてください。通勤、仕事、家事などの時間をすべて含めてお答えください。余暇は含めません。: 4.力のいる作業をしている時間** | | |
| なかった | 393 (40%) | 276 (34%) |
| 1時間未満 | 429 (44%) | 371 (46%) |
| 1時間以上3時間未満 | 94 (9.6%) | 101 (13%) |
| 3時間以上5時間未満 | 30 (3.1%) | 33 (4.1%) |
| 5時間以上7時間未満 | 18 (1.8%) | 12 (1.5%) |
| 7時間以上9時間未満 | 10 (1.0%) | 5 (0.6%) |
| 9時間以上11時間未満 | 0 (0%) | 2 (0.2%) |

[1] Note: Items shown in this table were presented in Japanese in the original survey.

[2] Median (Q1, Q3); n (%)

| 質問項目[1] | 女性<br>N = 975[2] | 男性<br>N = 801[2] |
|---|---|---|
| 11時間以上 | 1 (0.1%) | 1 (0.1%) |
| **あなたの現在の状況について、以下の質問にお答えください。: 1.必要な時に、あなたの話を聞いてくれる人がいますか** | | |
| ほとんどいない | 70 (7.2%) | 129 (16%) |
| たまにいる | 171 (18%) | 132 (16%) |
| ときどきいる | 206 (21%) | 185 (23%) |
| よくいる | 273 (28%) | 174 (22%) |
| いつでもいる | 255 (26%) | 181 (23%) |
| **あなたの現在の状況について、以下の質問にお答えください。: 2.なにか困ったことがあった時、よいアドバイスをくれる人がいますか** | | |
| ほとんどいない | 96 (9.8%) | 164 (20%) |
| たまにいる | 188 (19%) | 167 (21%) |
| ときどきいる | 233 (24%) | 209 (26%) |
| よくいる | 243 (25%) | 140 (17%) |
| いつでもいる | 215 (22%) | 121 (15%) |
| **あなたの現在の状況について、以下の質問にお答えください。: 3.あなたを心配したり、あなたに愛情をかけてくれる人はいますか** | | |
| ほとんどいない | 61 (6.3%) | 111 (14%) |
| たまにいる | 150 (15%) | 153 (19%) |
| ときどきいる | 192 (20%) | 190 (24%) |
| よくいる | 242 (25%) | 156 (19%) |
| いつでもいる | 330 (34%) | 191 (24%) |
| **あなたの現在の状況について、以下の質問にお答えください。: 4.日常の家事をしたり、手伝ってくれる人はいますか** | | |
| ほとんどいない | 213 (22%) | 156 (19%) |
| たまにいる | 150 (15%) | 101 (13%) |
| ときどきいる | 202 (21%) | 152 (19%) |
| よくいる | 192 (20%) | 164 (20%) |

[1] Note: Items shown in this table were presented in Japanese in the original survey.

[2] Median (Q1, Q3); n (%)

| 質問項目[1] | 女性<br>N = 975[2] | 男性<br>N = 801[2] |
|---|---|---|
| いつでもいる | 218 (22%) | 228 (28%) |
| **あなたの現在の状況について、以下の質問にお答えください。: 5.あなたに情緒的な支えを与えてくれるような人、（たとえば、あなたの直面する問題について相談できる人、難しい判断が必要な時に助けてくれる人）はいますか** | | |
| ほとんどいない | 105 (11%) | 180 (22%) |
| たまにいる | 154 (16%) | 116 (14%) |
| ときどきいる | 201 (21%) | 183 (23%) |
| よくいる | 240 (25%) | 155 (19%) |
| いつでもいる | 275 (28%) | 167 (21%) |
| **あなたの現在の状況について、以下の質問にお答えください。: 6.必要な時にいつでも連絡がとれる、親しくて、信頼・信用できる人はいますか** | | |
| ほとんどいない | 103 (11%) | 149 (19%) |
| たまにいる | 144 (15%) | 133 (17%) |
| ときどきいる | 180 (18%) | 193 (24%) |
| よくいる | 264 (27%) | 148 (18%) |
| いつでもいる | 284 (29%) | 178 (22%) |
| **あなたの現在の状況について、以下の質問にお答えください。: 1.気軽に個人的な相談ができる親しい友人は何人いますか** | | |
| 0人 | 225 (23%) | 282 (35%) |
| 1人 | 207 (21%) | 166 (21%) |
| 2人 | 258 (26%) | 159 (20%) |
| 3人以上 | 285 (29%) | 194 (24%) |
| **あなたの現在の状況について、以下の質問にお答えください。: 2.気軽に個人的な相談ができる親類は何人いますか** | | |
| 0人 | 195 (20%) | 221 (28%) |
| 1人 | 277 (28%) | 210 (26%) |
| 2人 | 258 (26%) | 174 (22%) |

[1] Note: Items shown in this table were presented in Japanese in the original survey.

[2] Median (Q1, Q3); n (%)

| 質問項目[1] | 女性<br>N = 975[2] | 男性<br>N = 801[2] |
|---|---|---|
| 3人以上 | 245 (25%) | 196 (24%) |
| **地域組織、自助集団、チャリティー、ボランティアグループや、宗教団体などの集まりにどれくらいの頻度で参加していますか。** | | |
| 全く／ほとんど参加しない | 858 (88%) | 678 (85%) |
| 時々参加する | 84 (8.6%) | 87 (11%) |
| 週に1回未満 | 12 (1.2%) | 16 (2.0%) |
| 週に1回以上 | 21 (2.2%) | 20 (2.5%) |
| **あなたは、生きがいがあると感じていますか。** | | |
| 非常にある | 148 (15%) | 101 (13%) |
| ある | 480 (49%) | 399 (50%) |
| あまりない | 273 (28%) | 219 (27%) |
| 全くない | 74 (7.6%) | 82 (10%) |
| **あなたはご自分がどれくらい幸せだと感じていますか。** | | |
| 大変幸せ | 158 (16%) | 100 (12%) |
| 幸せ | 539 (55%) | 394 (49%) |
| どちらとも言えない | 213 (22%) | 237 (30%) |
| 幸せでない | 65 (6.7%) | 70 (8.7%) |
| **最近1週間の体や心の状態について、お聞きします。それぞれ最もあてはまる選択肢1つ選択してください。: 1.食べたくない。食欲がおちた** | | |
| 全く／ほとんどなかった | 772 (79%) | 668 (83%) |
| たまにあった（1〜2日） | 154 (16%) | 90 (11%) |
| しばしばあった（3〜4日） | 23 (2.4%) | 27 (3.4%) |
| いつもあった（ほぼ毎日） | 26 (2.7%) | 16 (2.0%) |
| **最近1週間の体や心の状態について、お聞きします。それぞれ最もあてはまる選択肢1つ選択してください。: 2.ゆううつだ** | | |
| 全く／ほとんどなかった | 536 (55%) | 499 (62%) |
| たまにあった（1〜2日） | 300 (31%) | 203 (25%) |
| しばしばあった（3〜4日） | 83 (8.5%) | 66 (8.2%) |

[1] Note: Items shown in this table were presented in Japanese in the original survey.

[2] Median (Q1, Q3); n (%)

| 質問項目[1] | 女性<br>N = 975[2] | 男性<br>N = 801[2] |
|---|---|---|
| いつもあった（ほぼ毎日） | 56 (5.7%) | 33 (4.1%) |
| **最近1週間の体や心の状態について、お聞きします。それぞれ最もあてはまる選択肢1つ選択してください。: 3.何をするのも面倒だ** | | |
| 全く／ほとんどなかった | 440 (45%) | 445 (56%) |
| たまにあった（1〜2日） | 359 (37%) | 253 (32%) |
| しばしばあった（3〜4日） | 108 (11%) | 65 (8.1%) |
| いつもあった（ほぼ毎日） | 68 (7.0%) | 38 (4.7%) |
| **最近1週間の体や心の状態について、お聞きします。それぞれ最もあてはまる選択肢1つ選択してください。: 4.なかなか眠れない** | | |
| 全く／ほとんどなかった | 562 (58%) | 558 (70%) |
| たまにあった（1〜2日） | 294 (30%) | 180 (22%) |
| しばしばあった（3〜4日） | 83 (8.5%) | 45 (5.6%) |
| いつもあった（ほぼ毎日） | 36 (3.7%) | 18 (2.2%) |
| **最近1週間の体や心の状態について、お聞きします。それぞれ最もあてはまる選択肢1つ選択してください。: 5.生活について満足して過ごせる** | | |
| 全く／ほとんどなかった | 232 (24%) | 218 (27%) |
| たまにあった（1〜2日） | 230 (24%) | 162 (20%) |
| しばしばあった（3〜4日） | 245 (25%) | 227 (28%) |
| いつもあった（ほぼ毎日） | 268 (27%) | 194 (24%) |
| **最近1週間の体や心の状態について、お聞きします。それぞれ最もあてはまる選択肢1つ選択してください。: 6.一人ぼっちでさびしい** | | |
| 全く／ほとんどなかった | 710 (73%) | 633 (79%) |
| たまにあった（1〜2日） | 182 (19%) | 107 (13%) |
| しばしばあった（3〜4日） | 59 (6.1%) | 28 (3.5%) |
| いつもあった（ほぼ毎日） | 24 (2.5%) | 33 (4.1%) |
| **最近1週間の体や心の状態について、お聞きします。それぞれ最もあてはまる選択肢1つ選択してください。: 7.皆がよそよそしいと思う** | | |
| 全く／ほとんどなかった | 786 (81%) | 638 (80%) |
| たまにあった（1〜2日） | 130 (13%) | 122 (15%) |

[1] Note: Items shown in this table were presented in Japanese in the original survey.

[2] Median (Q1, Q3); n (%)

| **質問項目**[1] | **女性**<br>N = 975[2] | **男性**<br>N = 801[2] |
|---|---|---|
| しばしばあった（3〜4日） | 41 (4.2%) | 26 (3.2%) |
| いつもあった（ほぼ毎日） | 18 (1.8%) | 15 (1.9%) |
| **最近1週間の体や心の状態について、お聞きします。それぞれ最もあてはまる選択肢1つ選択してください。: 8.毎日が楽しい** | | |
| 全く／ほとんどなかった | 194 (20%) | 189 (24%) |
| たまにあった（1〜2日） | 300 (31%) | 270 (34%) |
| しばしばあった（3〜4日） | 288 (30%) | 207 (26%) |
| いつもあった（ほぼ毎日） | 193 (20%) | 135 (17%) |
| **最近1週間の体や心の状態について、お聞きします。それぞれ最もあてはまる選択肢1つ選択してください。: 9.悲しいと感じる** | | |
| 全く／ほとんどなかった | 568 (58%) | 527 (66%) |
| たまにあった（1〜2日） | 295 (30%) | 207 (26%) |
| しばしばあった（3〜4日） | 81 (8.3%) | 46 (5.7%) |
| いつもあった（ほぼ毎日） | 31 (3.2%) | 21 (2.6%) |
| **最近1週間の体や心の状態について、お聞きします。それぞれ最もあてはまる選択肢1つ選択してください。: 10.皆が自分を嫌っていると感じる** | | |
| 全く／ほとんどなかった | 762 (78%) | 636 (79%) |
| たまにあった（1〜2日） | 141 (14%) | 124 (15%) |
| しばしばあった（3〜4日） | 46 (4.7%) | 28 (3.5%) |
| いつもあった（ほぼ毎日） | 26 (2.7%) | 13 (1.6%) |
| **最近1週間の体や心の状態について、お聞きします。それぞれ最もあてはまる選択肢1つ選択してください。: 11.仕事が手につかない** | | |
| 全く／ほとんどなかった | 756 (78%) | 613 (77%) |
| たまにあった（1〜2日） | 155 (16%) | 149 (19%) |
| しばしばあった（3〜4日） | 50 (5.1%) | 27 (3.4%) |
| いつもあった（ほぼ毎日） | 14 (1.4%) | 12 (1.5%) |
| **あなたの気持ち、考えなどについてお伺いします。: 1.過去1か月、「人生での大切な事が自分の思うようにならない」と感じましたか** | | |
| 全くない | 278 (29%) | 222 (28%) |

[1] Note: Items shown in this table were presented in Japanese in the original survey.

[2] Median (Q1, Q3); n (%)

| 質問項目[1] | 女性<br>N = 975[2] | 男性<br>N = 801[2] |
|---|---|---|
| ほとんどない | 298 (31%) | 244 (30%) |
| ときどき | 303 (31%) | 251 (31%) |
| 頻繁に | 58 (5.9%) | 40 (5.0%) |
| とても頻繁に | 38 (3.9%) | 44 (5.5%) |
| **あなたの気持ち、考えなどについてお伺いします。: 2.過去1か月、「自分の問題を解決する能力に自信がある」と感じましたか** | | |
| 全くない | 186 (19%) | 132 (16%) |
| ほとんどない | 272 (28%) | 187 (23%) |
| ときどき | 375 (38%) | 324 (40%) |
| 頻繁に | 111 (11%) | 127 (16%) |
| とても頻繁に | 31 (3.2%) | 31 (3.9%) |
| **あなたの気持ち、考えなどについてお伺いします。: 3.過去1か月、「思うように物事がいっている」と感じましたか** | | |
| 全くない | 148 (15%) | 127 (16%) |
| ほとんどない | 220 (23%) | 176 (22%) |
| ときどき | 463 (47%) | 377 (47%) |
| 頻繁に | 124 (13%) | 95 (12%) |
| とても頻繁に | 20 (2.1%) | 26 (3.2%) |
| **あなたの気持ち、考えなどについてお伺いします。: 4.過去1か月、「多くの困難が山積みで自分の手に負えない」と感じましたか** | | |
| 全くない | 280 (29%) | 242 (30%) |
| ほとんどない | 360 (37%) | 285 (36%) |
| ときどき | 254 (26%) | 199 (25%) |
| 頻繁に | 51 (5.2%) | 51 (6.4%) |
| とても頻繁に | 30 (3.1%) | 24 (3.0%) |
| **あなたの周りの状況について、以下の質問にお答えください。: 1.一般的に、人は信用できると思いますか** | | |
| 全く思わない | 83 (8.5%) | 87 (11%) |

[1] Note: Items shown in this table were presented in Japanese in the original survey.

[2] Median (Q1, Q3); n (%)

| 質問項目[1] | 女性<br>N = 975[2] | 男性<br>N = 801[2] |
|---|---|---|
| あまり思わない | 355 (36%) | 312 (39%) |
| 思う | 503 (52%) | 367 (46%) |
| 非常によく思う | 34 (3.5%) | 35 (4.4%) |
| **あなたの周りの状況について、以下の質問にお答えください。: 2.多くの人は隙さえあれば、他の人を利用しようとするものだと思いますか** | | |
| 全く思わない | 78 (8.0%) | 68 (8.5%) |
| あまり思わない | 542 (56%) | 392 (49%) |
| 思う | 318 (33%) | 297 (37%) |
| 非常によく思う | 37 (3.8%) | 44 (5.5%) |
| **あなたの周りの状況について、以下の質問にお答えください。: 3.多くの場合、人は他の人の役に立とうとすると思いますか** | | |
| 全く思わない | 51 (5.2%) | 69 (8.6%) |
| あまり思わない | 338 (35%) | 347 (43%) |
| 思う | 560 (57%) | 364 (45%) |
| 非常によく思う | 26 (2.7%) | 21 (2.6%) |
| **学校教育はどのくらいまで受けられましたか。1つだけ選択してください。** | | |
| 中学校 | 11 (1.1%) | 21 (2.6%) |
| 高校 | 234 (24%) | 192 (24%) |
| 短大卒・専門学校・4年制大学中退 | 297 (30%) | 102 (13%) |
| 大学以上 | 427 (44%) | 479 (60%) |
| その他 | 6 (0.6%) | 7 (0.9%) |
| **現在の従事している職業は何ですか。** | | |
| 無職 | 66 (6.8%) | 119 (15%) |
| 主婦（主夫） | 257 (26%) | 5 (0.6%) |
| 専門的・技術的職業従事者 | 94 (9.6%) | 140 (17%) |
| 管理的職業従事者 | 6 (0.6%) | 95 (12%) |
| 事務従事者 | 226 (23%) | 112 (14%) |

[1] Note: Items shown in this table were presented in Japanese in the original survey.

[2] Median (Q1, Q3); n (%)

| 質問項目[1] | 女性<br>N = 975[2] | 男性<br>N = 801[2] |
|---|---|---|
| 販売従事者 | 48 (4.9%) | 34 (4.2%) |
| サービス職業従事者 | 108 (11%) | 72 (9.0%) |
| 保安職業従事者 | 0 (0%) | 12 (1.5%) |
| 農業漁業作業者 | 2 (0.2%) | 5 (0.6%) |
| 運輸・通信従事者 | 7 (0.7%) | 32 (4.0%) |
| 生産工程・労務作業者 | 26 (2.7%) | 56 (7.0%) |
| その他の職業 | 135 (14%) | 119 (15%) |
| **現在従事されているお仕事の雇用形態は何ですか。** | | |
| 正社員・職員 | 343 (53%) | 489 (72%) |
| 契約社員・職員 | 49 (7.5%) | 44 (6.5%) |
| 派遣社員・職員 | 29 (4.4%) | 15 (2.2%) |
| パート・アルバイト | 196 (30%) | 44 (6.5%) |
| 自営・経営者 | 35 (5.4%) | 85 (13%) |
| 回答なし | 323 | 124 |
| **現在の世帯年収（税込み）はどのくらいですか。** | | |
| 0〜299万円 | 211 (22%) | 148 (18%) |
| 300〜599万円 | 375 (38%) | 280 (35%) |
| 600〜899万円 | 231 (24%) | 197 (25%) |
| 900〜1,199万円 | 97 (9.9%) | 104 (13%) |
| 1,200〜1,499万円 | 38 (3.9%) | 37 (4.6%) |
| 1,500万円以上 | 23 (2.4%) | 35 (4.4%) |

[1] Note: Items shown in this table were presented in Japanese in the original survey.

[2] Median (Q1, Q3); n (%)
