# Supplementary material for: Physical, Mental, and Social Characteristics Associated With Happiness in Individuals With Schizophrenia in Japan: A Cross‐Sectional Study
Source: Neuropsychopharmacol Rep. 2025 Sep 4;45(3):e70045. doi: 10.1002/npr2.70045 (PMC12409470; doi:10.1002/npr2.70045)
Supplement: Supplementary file 2 — Figure S1: Sex‐ and Age‐adjusted Odds Ratios and 95% Confidence Intervals for Happiness in Participants with and without Schizophrenia. Figure S2: Age‐adjusted Odds Ratios and 95% Confidence Intervals for Happiness in Participants with and without Schizophrenia, Stratified by Sex. Figure S3: Sex‐adjusted Odds Ratios and 95% Confidence Intervals for Happiness in Participants with and without Schizophrenia, Stratified by Age. [file NPR2-45-e70045-s001.docx]

**Supplementary Material**

Figure S1. Sex- and Age-adjusted Odds Ratios and 95% Confidence Intervals for Happiness in Participants with and without Schizophrenia.

Figure S2. Age-adjusted Odds Ratios and 95% Confidence Intervals for Happiness in Participants with and without Schizophrenia, Stratified by Sex.

Figure S3. Sex-adjusted Odds Ratios and 95% Confidence Intervals for Happiness in Participants with and without Schizophrenia, Stratified by Age.

Figure S1. Sex- and Age-adjusted Odds Ratios and 95% Confidence Intervals for Happiness in Participants with and without Schizophrenia.

Adjusted OR, Sex- and Age-adjusted odds ratio.

† P < 0.05 for the difference in PRs (P_diff_) between participants with and without schizophrenia.

Figure S2. Age-adjusted Odds Ratios and 95% Confidence Intervals for Happiness in Participants with and without Schizophrenia, Stratified by Sex.

Adjusted OR, Age-adjusted odds ratio.

† P < 0.05 for the difference in PRs (P_diff_) between participants with and without schizophrenia.

‡ P < 0.05 for the interaction (P_int_) between sex and each variable among participants with schizophrenia.

Figure S3. Sex-adjusted Odds Ratios and 95% Confidence Intervals for Happiness in Participants with and without Schizophrenia, Stratified by Age.

Adjusted OR, Sex-adjusted odds ratio.

† P < 0.05 for the difference in PRs (P_diff_) between participants with and without schizophrenia.

‡ P < 0.05 for the interaction (P_int_) between age and each variable among participants with schizophrenia.
